# Myricetin antagonizes semen-derived enhancer of viral infection (SEVI) formation and influences its infection-enhancing activity

**DOI:** 10.1186/s12977-018-0432-3

**Published:** 2018-07-16

**Authors:** Ruxia Ren, Shuwen Yin, Baolong Lai, Lingzhen Ma, Jiayong Wen, Xuanxuan Zhang, Fangyuan Lai, Shuwen Liu, Lin Li

**Affiliations:** 1 Guangdong Provincial Key Laboratory of New Drug Screening, Guangzhou Key Laboratory of Drug Research for Emerging Virus Prevention and Treatment, School of Pharmaceutical Sciences, Southern Medical University, 1838 Guangzhou Avenue North, Guangzhou, 510515 Guangdong China; 2 Department of Pharmacy, The Seventh Affiliated Hospital of Sun Yat-sen University, Shenzhen, 518107 China

**Keywords:** HIV, Myricetin, Amyloid fibrils, SEVI, Synergistic antiviral effects

## Abstract

**Background:**

Semen is a critical vector for human immunodeficiency virus (HIV) sexual transmission and harbors seminal amyloid fibrils that can markedly enhance HIV infection. Semen-derived enhancer of viral infection (SEVI) is one of the best-characterized seminal amyloid fibrils. Due to their highly cationic properties, SEVI fibrils can capture HIV virions, increase viral attachment to target cells, and augment viral fusion. Some studies have reported that myricetin antagonizes amyloid β-protein (Aβ) formation; myricetin also displays strong anti-HIV activity in vitro.

**Results:**

Here, we report that myricetin inhibits the formation of SEVI fibrils by binding to the amyloidogenic region of the SEVI precursor peptide (PAP248–286) and disrupting PAP248–286 oligomerization. In addition, myricetin was found to remodel preformed SEVI fibrils and to influence the activity of SEVI in promoting HIV-1 infection. Moreover, myricetin showed synergistic effects against HIV-1 infection in combination with other antiretroviral drugs in semen.

**Conclusions:**

Incorporation of myricetin into a combination bifunctional microbicide with both anti-SEVI and anti-HIV activities is a highly promising approach to preventing sexual transmission of HIV.

**Electronic supplementary material:**

The online version of this article (10.1186/s12977-018-0432-3) contains supplementary material, which is available to authorized users.

## Background

Since the first cases of acquired immune deficiency syndrome (AIDS) were reported in 1981, more than 70 million people have been infected by human immunodeficiency virus (HIV), and approximately 1 million die of the disease annually [1]. Currently, unprotected sex remains the major route of HIV transmission, accounting for more than 80% of new HIV infections worldwide [2].

It has been shown that semen functions as a critical carrier of virus during sexual transmission [3]. Notably, amyloid fibrils in semen are considered to be responsible for the reduced efficacy of antiretroviral therapies (cART) in vivo because they facilitate virus attachment and internalization into cells [4]. A naturally occurring C-proximal proteolytic fragment of prostatic acid phosphatase (PAP) in semen, PAP248–286, has been reported to form aggregated amyloid fibrils (known as semen-derived enhancer of virus infection or SEVI) and to increase by several orders of magnitude the rate of HIV infection in vitro [5]. Because SEVI is highly cationic and the fibrils it forms are positively charged, it not only effectively captures HIV virions but also promotes viral attachment to target cells by neutralizing the inherent electrostatic repulsion between the negative charges on the surfaces of the virions and target cells [6]. Some other endogenous amyloid aggregates that increase HIV-1 infectivity, including the PAP N-proximal fragment (PAP85–120) and semenogelins (SEM1 and SEM2), have been identified in human semen [7, 8], which may explain the discrepancy between the low infectiousness of HIV in vitro and its observed efficient sexual transmission.

As amyloid species that are naturally abundant in semen, including SEVI, play a critical role in the spread of HIV, they represent a particularly attractive target for the development of molecules that can reduce spread of the virus. Theoretically, eliminating seminal amyloid fibrils by antagonizing fibril formation or enhancing the degradation of mature endogenous fibrils can massively reduce any viral infection enhancement [9–11]. This strategy might be advantageous because it targets host factors rather than the virus itself.

It is well known that the aggregation of proteins into amyloid fibrils is associated with fatal diseases, including Alzheimer’s disease, Parkinson’s disease and diabetes, and many molecules that inhibit fibrillization in vitro have been reported. For instance, epigallocatechin-3-gallate (EGCG), the major catechin present in green tea, not only potently inhibits the amyloidogenesis of various polypeptides but is also able to disassemble a wide range of preformed amyloid fibrils [12, 13].

Myricetin, a common dietary flavonoid found in foods such as walnuts, onions, berries, herbs and red grapes, exerts a wide variety of biological and nutraceutical effects, including antioxidative, anti-inflammatory, antidiabetic, antitumor and free radical-scavenging activities [14–16]. Moreover, myricetin shows activity against HIV-1 infection by inhibiting HIV-1 integrase [17, 18]. It is noteworthy that myricetin inhibits amyloid fibrillization by a variety of disease-associated amyloidogenic proteins, including amyloid β-protein (Aβ), tau protein, islet amyloid polypeptide and other amyloidogenic peptides [19–21]. As described above, SEVI is a possible drug target for preventing HIV sexual transmission due to its stable structure and high levels in semen [22]. The concentration of endogenous SEVI in pooled semen is approximately 35 µg/ml, which considerably exceeds the level (≥ 2 µg/ml) required to enhance infection [7, 23]. However, it is not known whether myricetin affects the formation of SEVI amyloid fibrils in semen. In the current study, we sought to elucidate the effects of myricetin on the formation of SEVI amyloid fibrils and on the enhancement of HIV-1 infection by SEVI. Strikingly, the results showed that myricetin influences the activity of SEVI in enhancing HIV-1 infection and that it exhibits synergistic effects in semen against HIV-1 infection when applied to cells in combination with other antiretroviral (ARV) agents. These findings will be helpful in the development of dual-function antiviral drugs or microbicides that possess both anti-HIV and anti-SEVI-enhancing activities.

## Results

### Myricetin inhibits the formation of SEVI fibrils and other seminal fibrils

**Fig. 1:**
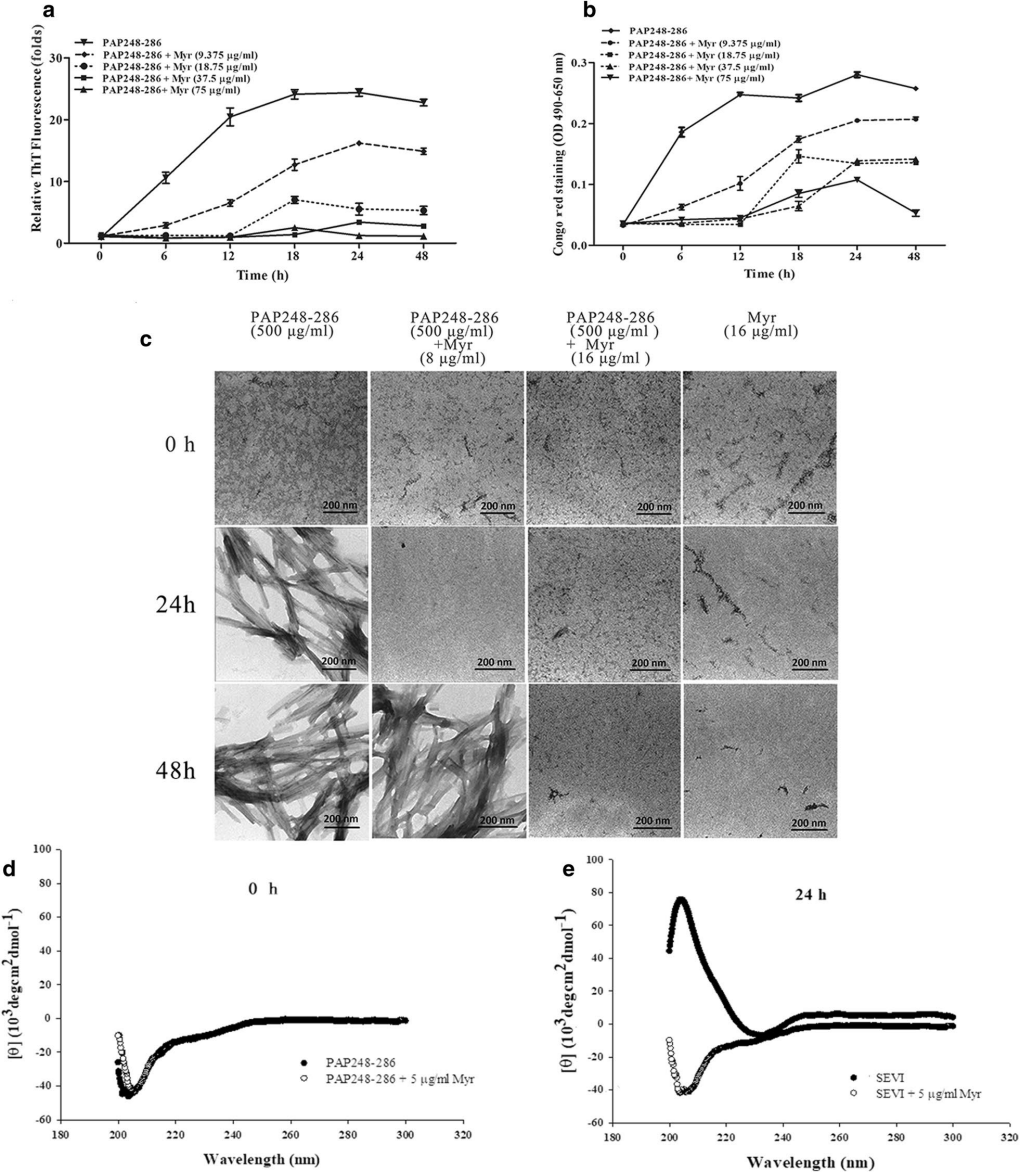
Myricetin inhibits the assembly of PAP248–286. PAP248–286 (3 mg/ml) was incubated with myricetin (75, 37.5, 18.75, 9.375 and 0 μg/ml), and the mixture was agitated at 1400 rpm at 37 °C. Samples were collected at various time points (0, 6, 12, 18, 24 and 48 h) and monitored by ThT (**a**) or Congo red staining (**b**). Average values (± SD) were calculated from triplicate measurements; the data shown represent one representative trial of three independent experiments. **c** Amyloid fibril samples (500 μg/ml) in the presence or absence of myricetin (8 and 16 μg/ml) at various time points (0, 24 and 48 h), as visualized by TEM. The scale bar is 200 nm. **d**, **e** The secondary structure of PAP248–286 or SEVI fibrils (300 μg/ml) in the presence or absence of myricetin (5 μg/ml) at various time points (0 and 24 h) was measured using CD spectroscopy

Thioflavin T (ThT), a dye that is commonly used in the detection of amyloid fibrils, can intercalate into the β-sheet structure of amyloid fibrils, resulting in enhanced fluorescence and a characteristic redshift in the emission spectrum. Congo red, another amyloid-binding dye, exhibits apple-green birefringence under polarized light as well as increased fluorescence [7, 24]. To evaluate the effects of myricetin on the formation of SEVI fibrils by PAP248–286, we collected PAP248–286 aggregates formed in the presence or absence of various concentrations of myricetin at different time points and monitored them using both ThT fluorescence and Congo red staining assays. The results showed that myricetin antagonized the assembly of PAP248–286 monomers into SEVI fibrils in a dose-dependent manner. The addition of myricetin at 75 µg/ml increased the lag time of SEVI amyloid fibril formation by PAP248–286 to more than 48 h, as measured by ThT fluorescence (Fig. 1a), whereas the lag time of amyloid fibril formation by PAP248–286 in the absence of myricetin was less than 6 h. Congo red assays showed that the lag time of SEVI amyloid fibril formation in the presence of various concentrations of myricetin was extended to approximately 18 h (Fig. 1b). The morphology of SEVI fibrils in the presence and absence of myricetin was imaged by transmission electron microscopy (TEM). As shown in Fig. 1c, mature SEVI fibrils formed by PAP248–286 were identified after shaking for 24 h, whereas no amyloid fibrils were observed after adding 16 μg/ml of myricetin, even after shaking for 48 h.

Far-UV (190–260 nm) circular dichroism (CD) spectrum measurement is commonly used to detect the presence of the typical secondary β-sheet structure of fibrils; such fibrils display a spectrum with a minimum at approximately 218 nm. A characteristic β-sheet secondary structure undergoes higher-level association to form protein aggregates and mature fibrils. Thus, we further investigated the secondary structure of SEVI fibrils formed in the presence or absence of myricetin by measuring their CD spectra. As illustrated in Fig. 1e, the spectrum of PAP248–286 after agitation for 24 h displayed a single minimum at approximately 230 nm, indicating the presence of a typical β-sheet component. However, the spectrum of PAP248–286 after agitation for 24 h in the presence of 5 μg/ml myricetin (final concentration) indicated the presence of a characteristic random coil structure, suggesting that PAP248–286 did not adopt any ordered or stable conformation and indicating that β-sheet aggregation had not occurred.

**Fig. 2:**
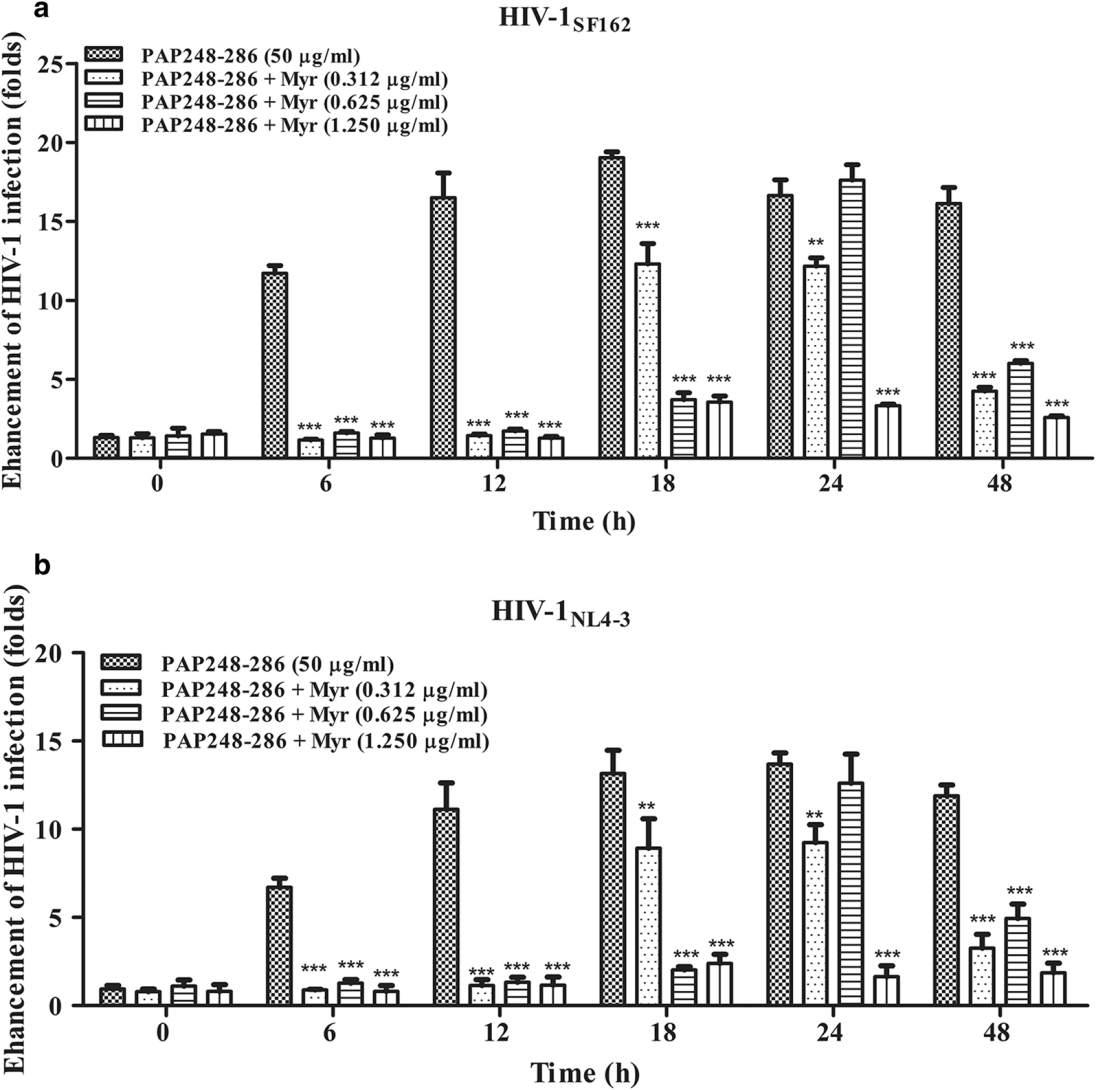
Amyloid fibril samples display loss of enhancement of HIV-1 infection in the presence of myricetin. Mixed SEVI fibril samples prepared as described above in the presence or absence of myricetin were diluted to a final concentration of 50 μg/ml (PAP248–286). The samples were then incubated with HIV-1_SF162_ (**a**) and HIV-1_NL4-3_ (**b**) infectious clones. The mixtures were added to prepared TZM-b1 cells. Luciferase activities were measured at 72 h post-infection. The values shown represent the mean ± SD (n = 3). One-way ANOVA with Dunnett’s post hoc multiple comparisons test was used to statistically analyze the differences between samples containing PAP248–286 alone and samples containing PAP248–286 and myricetin (*p < 0.05; **p < 0.01, ***p < 0.001)

Several studies have reported that the PAP248–286 monomer lacks the ability to promote HIV-1 infection [25]. To determine whether enhancement of HIV-1 infection by PAP248–286 is lost after the addition of myricetin, we characterized the HIV-1 infection-enhancing activity of PAP248–286 samples after agitation in the presence or absence of myricetin for various times (samples in Fig. 1a, b); the final concentrations of PAP and myricetin used in the cell-based HIV infection assay are only one-sixtieth of their initial concentrations. To eliminate the anti-HIV activity of myricetin itself, the above samples were first centrifuged at 12,000 rpm for 5 min to remove free myricetin. As shown in Fig. 2 and Additional file 1: Figure S2, PAP248–286 that had been agitated for 6–48 h effectively enhanced HIV-1 infection by two HIV-1 clones in a time-dependent manner. However, the ability of PAP248–286 to enhance HIV-1 infection decreased after agitation in the presence of myricetin in a dose-dependent manner. The highest final concentration of myricetin tested (1.25 µg/ml) significantly neutralized SEVI-mediated enhancing activity by inhibiting the formation of SEVI fibrils (Fig. 2, Additional file 1: Figure S2). It is worth mentioning that inhibition of SEVI fibril formation was not observed after agitation of PAP248–286 in the presence of lower concentrations of myricetin for 24 h but was observed after agitation of PAP248–286 in the presence of the same concentrations of myricetin for 48 h (Fig. 2). The most likely reason for this is that the ability of myricetin to inhibit fibril formation may require long exposure times and full interaction. The results also indicated that myricetin may play a role in the degradation of mature SEVI fibrils. Taken together, these findings show that myricetin abrogates the formation of SEVI fibrils by PAP248–286, thereby influencing the activity of SEVI in enhancing HIV-1 infection.

The effects of myricetin on the formation of two other common seminal amyloid fibrils, SEM1 and SEM2, were also assessed. According to the results, myricetin also inhibited the formation of fibrils by both SEM1_86–107_ and SEM2_86–107_ in a dose-dependent manner (Additional file 1: Figure S1). The addition of myricetin at a concentration of 200 µg/ml completely blocked amyloid fibril formation, as measured by ThT fluorescence.

### Myricetin shows binding affinity to functional regions of the PAP248–286 monomer

**Fig. 3:**
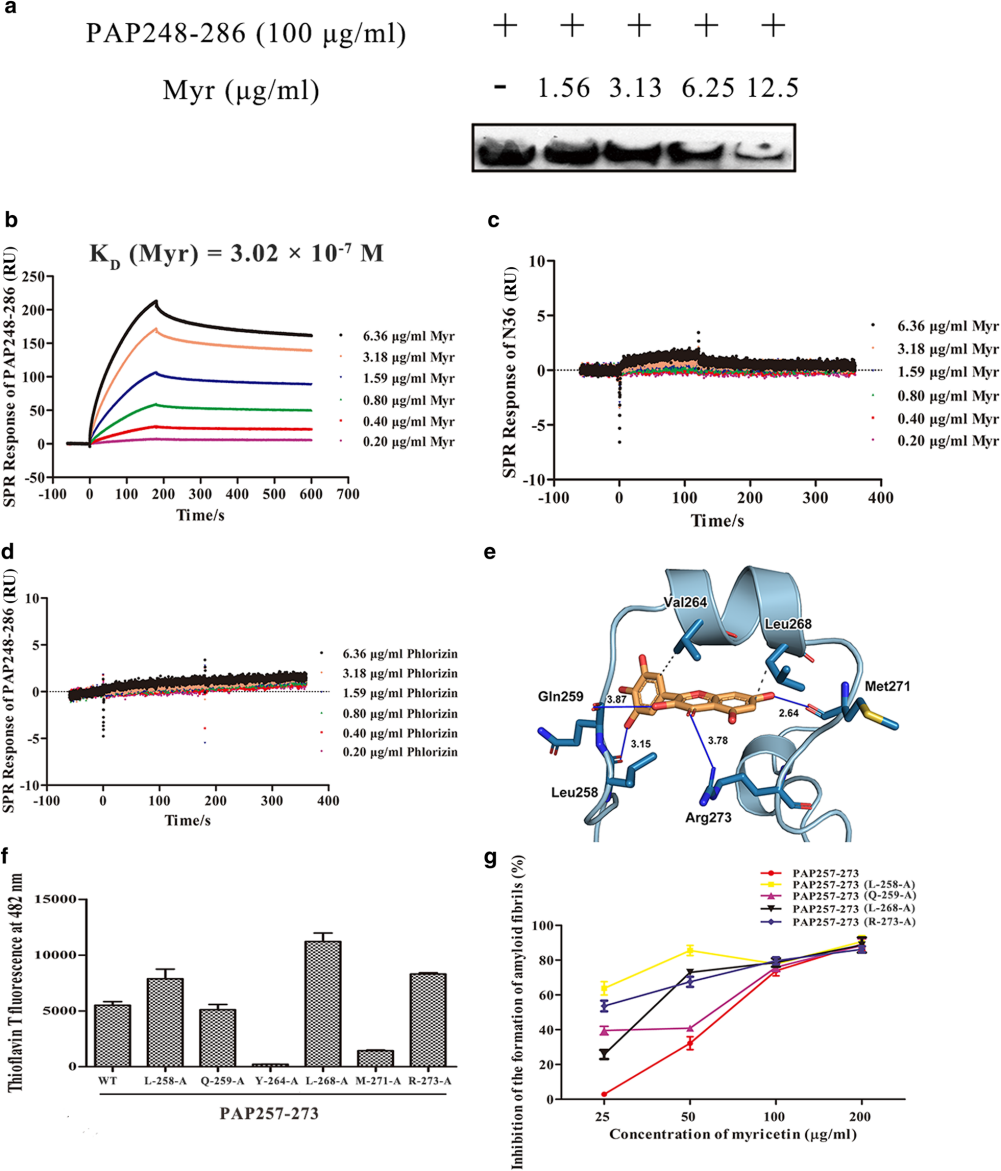
Myricetin exhibits high-affinity binding to the functional region of the PAP248–286 monomer. **a** The electrostatic interaction of myricetin with PAP248–286. PAP248–286 (100 μg/ml) was incubated with serially diluted myricetin (12.5, 6.25, 3.13 and 1.56 μg/ml) at 37 °C for 30 min. The samples were then centrifuged at 5000 rpm for 15 min; the peptides remaining in the supernatant were electrophoresed through 10% acidic native polyacrylamide gels and detected by WB using a polyclonal antibody against PAP248–286. **b** Binding affinity of myricetin to PAP248–286. PAP248–286 (50 μg/ml) was immobilized on a sensor chip. Subsequently, twofold serial dilutions of recombinant myricetin were injected as the analyte. The affinity constant *K*_*D*_ is the ratio of the dissociation constant *K*_*d*_ to the association constant *K*_*a*_ (*K*_*D*_= *K*_*d*_/*K*_*a*_). **c** Binding affinity of myricetin to an irrelevant N36 peptide (50 μg/ml). **d** Binding affinity of an irrelevant flavonoid (phlorizin) to PAP248–286 (50 μg/ml). **e** Presumed binding sites of myricetin to PAP248–286. According to the computational docking results, myricetin formed hydrogen bonds with Leu258, GIn259, Met271 and Arg273, and it bound to Val264 and Leu268 by hydrophobic interactions. **f** Amyloid fibril formation by wild-type PAP257–273 and six mutants. Wild-type and mutant peptides (3 mg/ml) were agitated at 1400 rpm at 37 °C for 48 h; the presence of fibrils was then monitored using ThT assays. **g** Myricetin inhibition of fibril formation by wild-type and mutants PAP257–273. Wild-type and mutants PAP257–273 were mixed with myricetin (200, 100, 50 and 25 μg/ml), and the mixtures were agitated at 1400 rpm at 37 °C. The samples were then collected and monitored using ThT assays. Percent inhibition was calculated from the following equation: (1-ThT fluorescence of fibrils with Myr/ThT fluorescence of fibrils without Myr) × 100. Average values (± SD) were calculated from triplicate measurements; the data shown represent one representative trial of three independent experiments

To clarify the molecular mechanism by which myricetin inhibits assembly of PAP248–286 into fibrils, we first analyzed potential interaction between myricetin and the PAP248–286 monomer by western blotting (WB). Myricetin is a negatively charged compound, whereas PAP248–286 is positively charged peptide. The inherent electrostatic repulsion that exists between two oppositely charged molecules might account for deposition of the PAP248–286 peptide via a change in isoelectric point. Using native acid gel electrophoresis, we observed a marked decrease in the level of PAP248–286 monomer in the supernatant after adding myricetin, a decrease that was dose dependent (Fig. 3a). The results indicate that myricetin binds to PAP248–286 monomers and forms insoluble aggregates via natural electrostatic interaction. The affinity between myricetin and the PAP248–286 monomer was further investigated using surface plasmon resonance (SPR). As shown in Fig. 3b, myricetin bound to the PAP248–286 monomer with high affinity (*K*_*D*_ = 3.02 × 10^−7^ M). To confirm the specificity of this binding, an irrelevant peptide (N36) and an irrelevant flavonoid (phlorizin) were used as negative controls; N36 is a 36-mer synthetic peptide derived from the N-terminal heptad repeat region of the HIV gp41 envelope protein [26], and phlorizin is a natural phenol that is found in apple and pear tree leaves. Our results confirmed that myricetin does not bind to N36 and that phlorizin does not bind to the PAP248–286 monomer (Fig. 3c, d).

To further substantiate the interaction of myricetin with PAP248–286 at the atomic level, computational molecular docking was conducted to identify the potential amino acids involved in the binding of myricetin to PAP248–286 monomers. Our analysis, which was based on the 3D molecular structure of myricetin and the monocrystal PAP248–286 protein structure reported in Protein Data Bank (PDB; code No. 2L3H), showed that myricetin is able to bind to PAP248–286 and form hydrogen bonds via four potential residues: Leu258, GIn259, Met271 and Arg273. Myricetin is also able to bind to PAP248–286 via hydrophobic interactions with Val264 and Leu268 (Fig. 3e).

Some studies have reported that the central region of the SEVI precursor peptide PAP248–286, namely, PAP257–267, is an amyloidogenic region with high fibril-forming propensity [27]. Coincidentally, PAP268–271 plays a vital role in promoting SEVI fibrillation [27]. According to our computational molecular docking results, six potential residues of PAP248–286 are associated with myricetin binding (Fig. 3e). To replace PAP248–286, we synthesized a 15-amino acid PAP257–271 fragment that includes both of the amyloidogenic regions (PAP257–267 and PAP268–271) and all six of the amino acids (Leu258, GIn259, Val264, Leu268, Met271 and Arg273) shown to be important in the molecular docking analysis. Importantly, due to its short length, the PAP257–271 peptide is simpler to synthesize on a large scale than is the SEVI peptide. In this study, we confirmed that the wild-type PAP257–273 fragment readily forms amyloid fibrils (Fig. 3f). To investigate whether the above-named six residues are essential for the formation of SEVI fibrils, we assessed the aggregation of PAP257–273 peptides containing site-directed mutations of these residues. As shown in Fig. 3f, four mutants of PAP257–273, i.e., Leu258A, GIn259A, Leu268A and Arg273A, exhibited fibril-forming propensity (Fig. 3f). Notably, PAP257–273 peptides with Val264A and Met271A mutations displayed attenuated aggregation during fibril formation, indicating that Val264 and Met271 of PAP248–286 might be critical for the assembly of PAP248–286 into fibrils. The inhibitory effects of myricetin on fibril formation by the four mutants of PAP257–273 were similar to or even stronger than their effects on wild-type PAP257–273, as measured by ThT assays (Fig. 3g).

### Myricetin inhibits oligomerization of PAP248–286 monomers

**Fig. 4:**
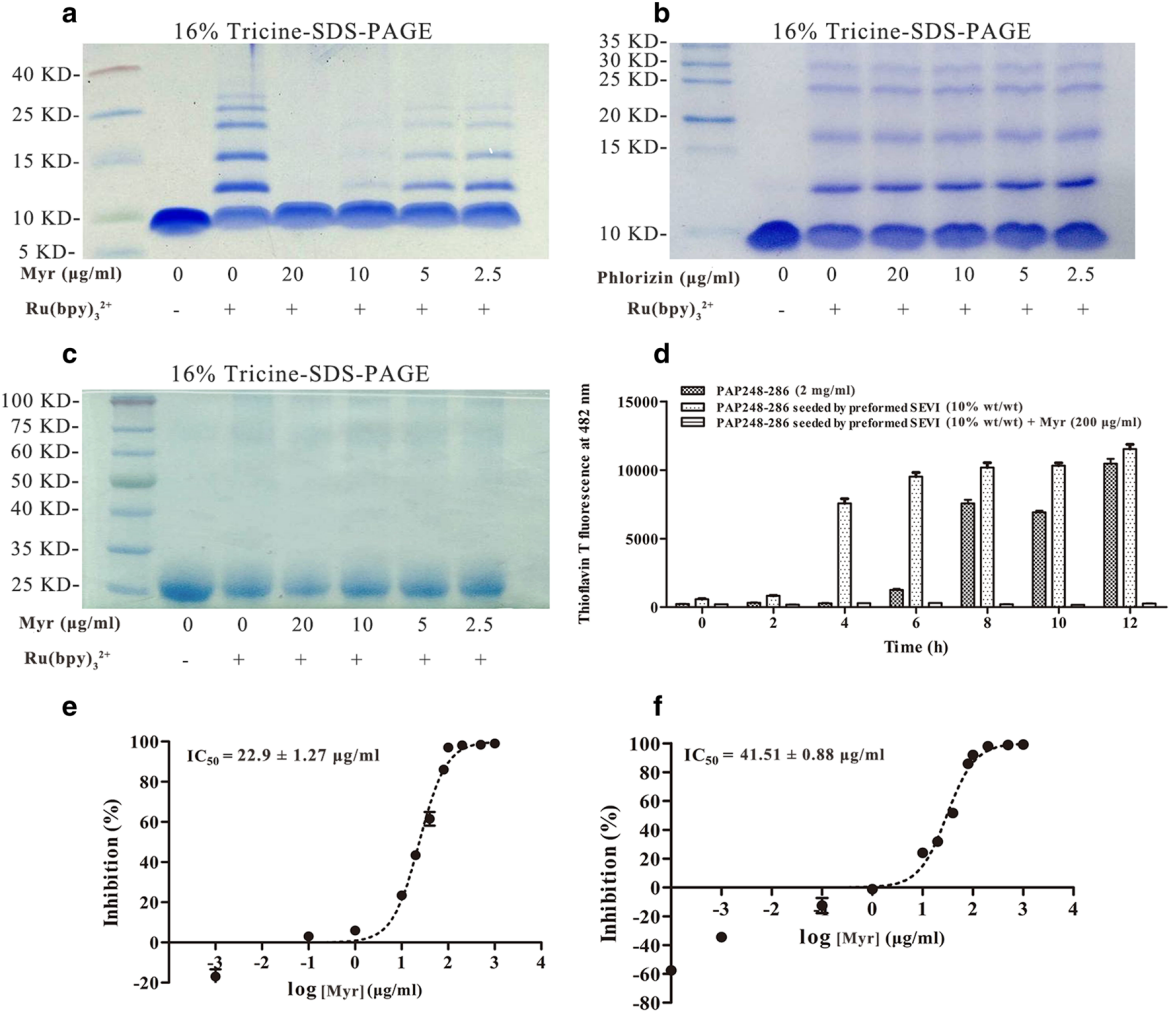
Myricetin inhibits early oligomerization, fibril extension and degradation of PAP248–286. **a** The effects of myricetin on the oligomerization of PAP248–286, as assessed using 16% Tricine-SDS-PAGE and Coomassie blue staining. PAP248–286 monomer at 500 μg/ml with or without cross-linking served as the control. **b** The effects of an irrelevant flavonoid (phlorizin) on the oligomerization of PAP248–286. **c** The effects of myricetin on the oligomerization of GST. **d** The effects of myricetin on fibril extension by PAP248–286. Mature SEVI fibril seeds (1% wt/wt) were mixed with PAP248–286 peptide (2 mg/ml) in the presence or absence of myricetin (200 μg/ml), and the mixture was agitated at 1400 rpm at 37 °C. Aliquots were collected at various time points and assessed by ThT fluorescence. **e** Dose–response curve for myricetin inhibition of PAP248–286 (2 mg/ml) fibrillization after 48 h of agitation by ThT assay. **f** Dose–response curve for myricetin inhibition of PAP248–286 (2 mg/ml) fibrillization seeded by preformed SEVI fibrils (10% wt/wt) after 48 h of agitation by ThT assay. IC_50_ values were calculated. The values shown represent the mean ± SD (n = 3)

Considerable evidence shows that the presence of low-*n*-order oligomers during the early stages of fibril formation indicates a lag phase in fibril assembly. It has been reported that myricetin inhibits Aβ aggregation by preventing amyloid β-protein oligomerization [28]. We therefore used photo-induced cross-linking of unmodified proteins (PICUP) to determine whether myricetin inhibits PAP248–286 monomer oligomerization and found that myricetin significantly inhibited oligomerization of PAP248–286 monomers in a dose-dependent manner (Fig. 4a). When 20 μg/ml of myricetin was mixed with 500 μg/ml of PAP248–286, oligomerization did not proceed to completion. In the presence of cross-linking agents, the PAP248–286 monomer, which was used as a positive control, predominantly existed as a mixture of oligomers on the order of 2–7. An irrelevant protein (glutathione S-transferase, GST) and an irrelevant flavonoid (phlorizin) were used as negative controls; GST normally exists as a mixture of homodimers and higher-order cross-linked species, thus providing an appropriate positive control for cross-linking chemistry. The results showed that phlorizin did not inhibit early oligomerization of PAP248–286 and that myricetin did not block oligomerization of GST (Fig. 4b, c).

### Myricetin antagonizes assembly of SEVI seeded by preformed fibrils

It has been reported that the addition of a small amount of preformed SEVI fibrils as seeds to a suspension of soluble PAP248–286 monomers promotes fibril polymerization and eliminates the lag phase for assembly [23, 29]. In the present study, myricetin-mediated inhibition of SEVI aggregation seeded by preformed fibrils was determined by the ThT assay. In contrast to unseeded PAP248–286 solutions, PAP248–286 solutions seeded with preformed SEVI fibrils showed rapid assembly into fibrils (Fig. 4d). In addition to antagonizing unseeded PAP248–286 assembly (Fig. 1a, b), myricetin also completely obstructed SEVI-seeded fibrillization (Fig. 4d), indicating that myricetin antagonizes the growth and aggregation of SEVI fibrils after nucleation. ThT assay results were utilized to generate a dose–response curve for myricetin inhibition of PAP248–286 (2 mg/ml) seeded or not with 10% wt/wt mature SEVI fibrils agitated for 48 h (Fig. 4e, f), and we obtained a half maximal inhibitory concentration (IC_50_) value of 41.51 μg/ml for myricetin inhibition of seeded PAP248–286 assembly compared to an IC_50_ value of 22.59 μg/ml for myricetin inhibition of spontaneous PAP248–286 assembly. Thus, higher concentrations of myricetin (200 μg/ml) were required to inhibit PAP248–286 assembly after the addition of SEVI seeds (Fig. 4d–f).

### Myricetin remodels mature SEVI fibrils

**Fig. 5:**
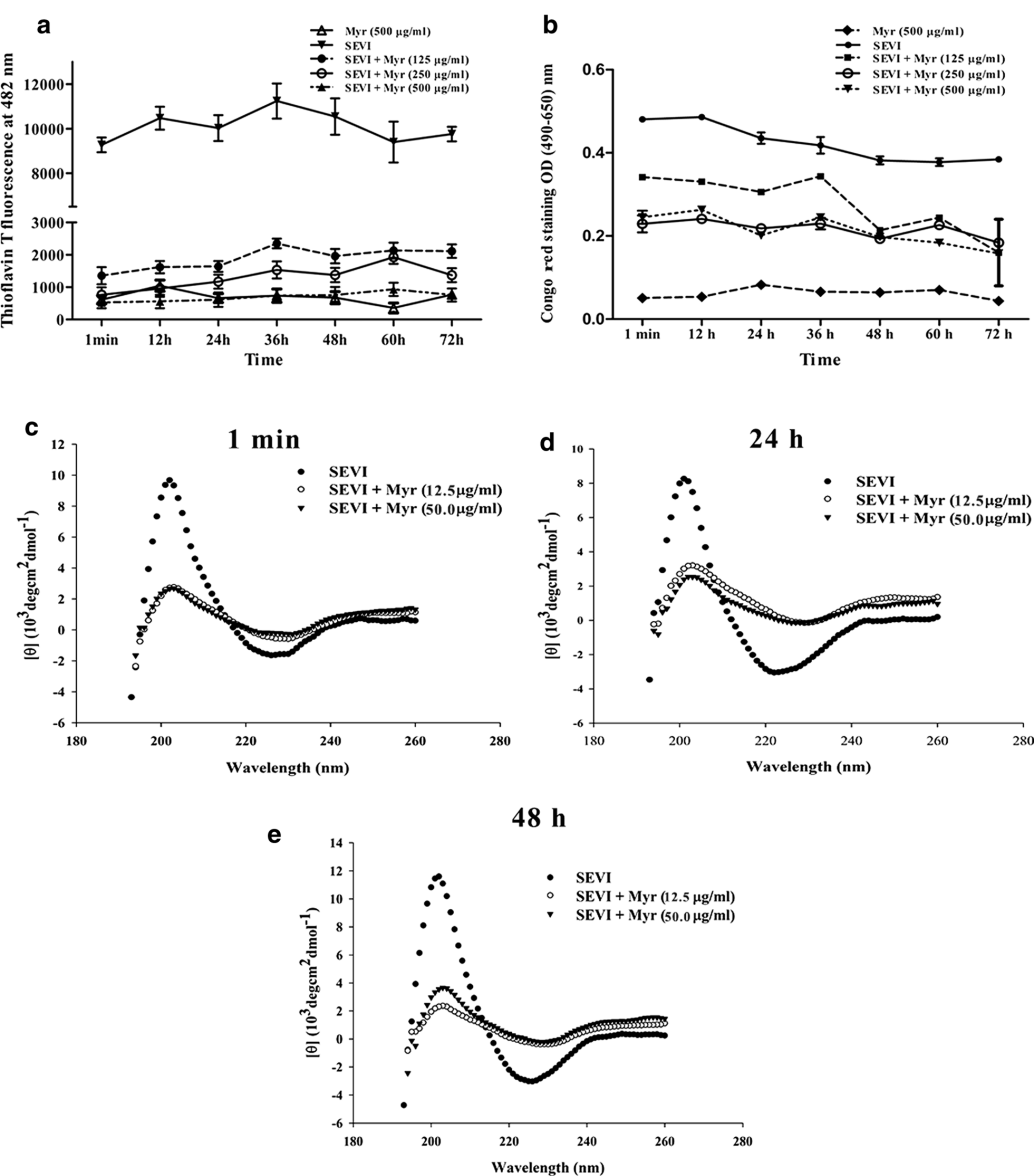
Remodeling of fibrils was monitored using ThT (**a**) or Congo red (**b**) assays. Mature SEVI fibrils were incubated with various concentrations of myricetin (500, 250, 125 μg/ml) or PBS and agitated as described above for 72 h. Aliquots were removed at the indicated time points. **c**–**e** At various time points (1 min, 24 and 48 h), the samples collected in the above-described remodeling experiment were measured by CD. Average values (± SD) were calculated from triplicate measurements; the data shown represent one representative trial of three independent experiments

ThT and Congo red assays were employed to determine whether myricetin can disassemble preformed fibrils, and the results showed that myricetin remodeled mature SEVI fibrils immediately in a dose-dependent but not in a time-dependent manner (Fig. 5a, b). Similar results showing that myricetin leads to a decrease in the β-sheet minimum of SEVI fibrils were obtained by CD, both immediately at 1 min and up to 48 h (Fig. 5c–e).

### Myricetin influences the activity of SEVI in enhancing HIV-1 infection

**Fig. 6:**
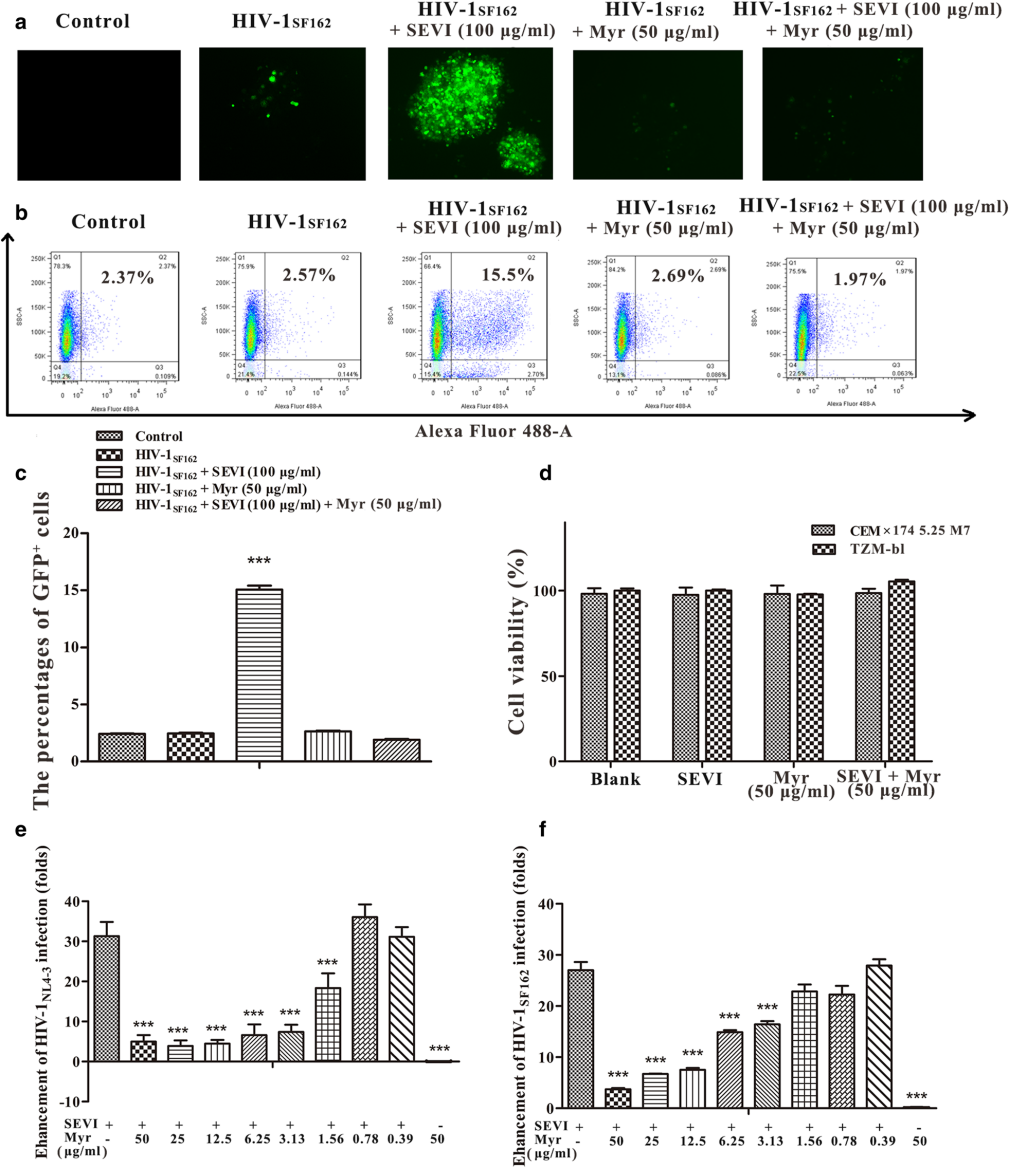
Myricetin reduces the activity of SEVI in enhancing HIV-1 infection. CEMx174 5.25M7 cells infected by HIV-1 _SF162_ in the presence or absence of SEVI (100 μg/ml) and myricetin (50 μg/ml) were imaged by fluorescence microscopy (**a**) and flow cytometric analysis (**b**). **c** The percentages of GFP^+^ CEMx174 5.25 M7 cells from multiple experiments. **d** Cell viability assay of CEMx174 5.25 M7 and TZM-bl cells with or without SEVI (100 or 50 μg/ml) or/and myricetin (50 μg/ml). SEVI (50 μg/ml) was incubated with myricetin at various concentrations (50, 25, 12.5, 6.25, 3.13, 1.56, 0.78 and 0.39 μg/ml). The mixtures were centrifuged to remove soluble myricetin. The pellets were resuspended in the original volume of medium and mixed with CCR5-tropic HIV-1_SF162_ (**e**) or CXCR4-tropic HIV-1_NL4-3_ (**f**). The luciferase activities of the cultures were measured at 72 h post-infection. Average values (± SD) were calculated from triplicate measurements; the data shown represent one representative trial of three independent experiments. One-way ANOVA with Dunnett’s post hoc multiple comparisons test was used to statistically analyze differences between samples containing SEVI alone and samples containing SEVI and myricetin (*p < 0.05; **p < 0.01, ***p < 0.001)

The effects of myricetin on the SEVI-mediated enhancement of HIV-1 infection were assessed by fluorescence microscopy, flow cytometry and HIV-1 infection assays. CEMx174 5.25 M7 cells expressing a green fluorescent protein (GFP) reporter gene under the control of the HIV-1 promoter were chosen as target cells. Fluorescence microscopy images revealed faint background GFP^+^ fluorescence expression in uninfected CEMx174 5.25 M7 cells and that SEVI fibril formation increased the fluorescence intensity of HIV-1-infected cells. However, myricetin notably decreased the fluorescence intensity of HIV-1-infected cells in the presence of SEVI fibrils (Fig. 6a). Subsequent flow cytometry assays further confirmed that myricetin reduced the number of cells infected by HIV-1 in the presence of SEVI fibrils (Fig. 6b). When SEVI fibrils were added to cells, the percentage of GFP^+^ CEMx174 5.25 M7 cells was increased to 15.5%, whereas the initial percentage of GFP^+^ cells of the control group in the absence of SEVI fibrils and HIV-1_SF162_ was 2.37%. The percentage of GFP^+^ cells of the HIV-1_SF162_ group without SEVI fibrils was 2.57%, though the percentage of GFP^+^ cells decreased to 1.97% upon incubation with myricetin (Fig. 6b). Similar to the HIV-1_SF162_ control, the initial percentage of GFP^+^ cells after incubation with HIV-1_SF162_ and myricetin in the absence of SEVI fibrils was approximately 2.69%. To demonstrate the reproducibility of the results, additional bar graph showing the averaged data of the percentages of GFP^+^ CEMx174 5.25 M7 cells from multiple experiments is presented in Fig. 6c.

To directly measure the effects of myricetin on SEVI-mediated enhancement of HIV-1 infection, we performed HIV-1 infection assays in the presence of SEVI fibrils. To eliminate the anti-HIV activity of myricetin itself as much as possible, we removed free myricetin by washing myricetin-treated SEVI samples one to five times with phosphate-buffered saline (PBS) followed by centrifugation prior to the assays. In contrast to samples containing only SEVI fibrils, myricetin-treated SEVI fibrils after one wash resulted in significant attenuation in infectivity of CCR5-tropic HIV-1_SF162_ and CXCR4-tropic HIV-1_NL4-3_ infectious clones in a dose-dependent manner (Fig. 6e, f) [7]. Our results further showed that myricetin-mediated inhibition of SEVI-mediated enhancement of HIV infection was still observed after five washes (Additional file 1: Figure S3).

We further assessed the viability of on both CEMx174 5.25 M7 and TZM-bl cells after exposure to myricetin at the highest concentration in parallel with all infection assays under the exact same conditions. As shown in Fig. 6d, myricetin at 50 μg/ml exhibited no cytotoxicity toward CEMx174 5.25 M7 and TZM-b1 cells, with almost 100% cell viability being observed.

### Interaction of SEVI with virions is affected by myricetin [30]

**Fig. 7:**
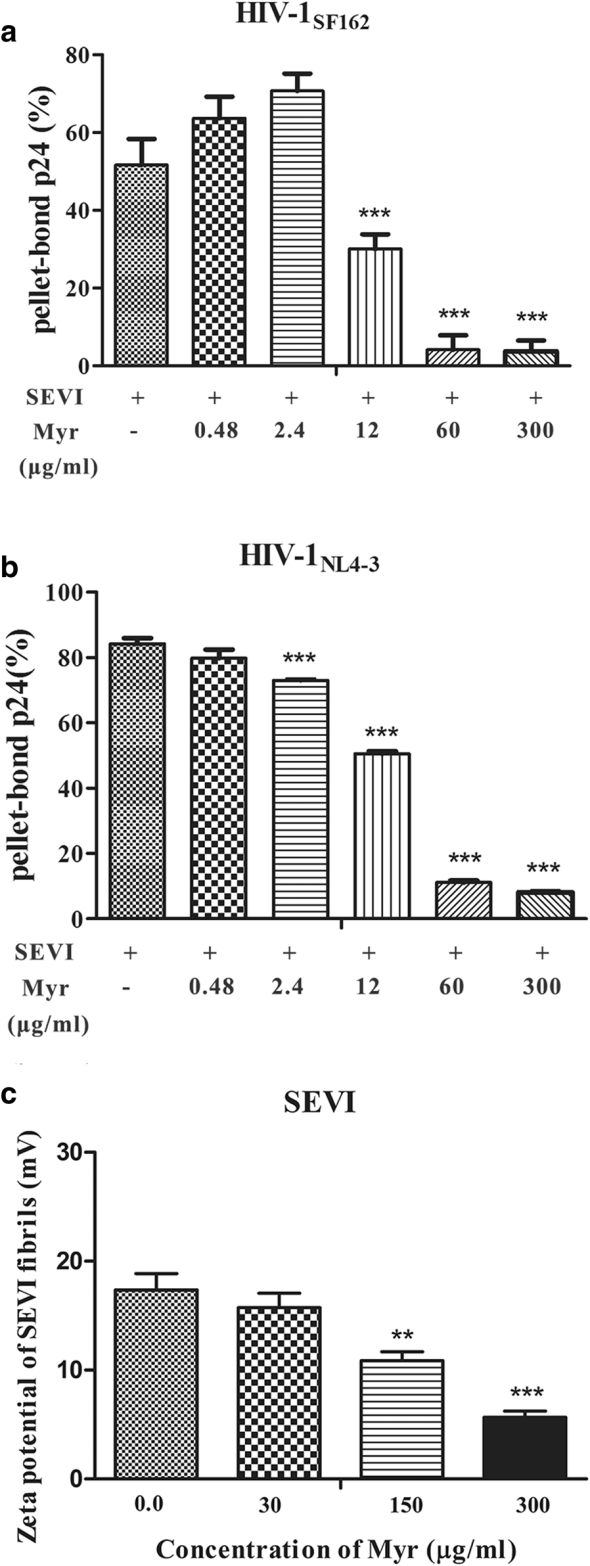
Myricetin blocks the interaction of SEVI with HIV-1 virions. SEVI samples (200 μg/ml) were first incubated with graded concentrations of myricetin for 30 min. The samples were then centrifuged at 12,000 rpm for 5 min; the supernatant was removed, and the pellets were resuspended and mixed with HIV-1_SF162_ (**a**) and HIV-1_NL4-3_ (**b**). These mixtures were centrifuged, and the p24 antigens present in the pellet were evaluated using a p24-antigen ELISA. **c** The zeta potential of SEVI fibrils in the presence or absence of myricetin was measured using Zeta Nanosizer. Average values (± SD) were calculated from triplicate measurements; the data shown represent one representative trial of three independent experiments. One-way ANOVA with Dunnett’s post hoc multiple comparisons test was used to statistically analyze the differences between samples containing SEVI alone and samples containing SEVI and myricetin (*p < 0.05; **p < 0.01, ***p < 0.001)

The effects of myricetin on the virus-binding ability of SEVI were examined using a virus pull-down assay. The results showed that SEVI fibrils alone bound to more than 51.6% of the input HIV-1 virions, including HIV-1_SF162_ and HIV-1_NL4-3_ infectious clones. Myricetin significantly abrogated the binding of all tested HIV-1 virions to SEVI fibrils in a dose-dependent manner (Fig. 7a–c), indicating that myricetin influences SEVI’s activity in enhancing HIV-1 infection by preventing the formation of virion-amyloid complexes. The positive surface charge of SEVI fibrils facilitates virion attachment to target cells and enhances HIV infection by serving as a polycationic bridge [7]. Thus, we determined whether negatively charged myricetin attenuates the interaction of SEVI with HIV-1 virions by affecting the cationic properties of SEVI fibrils. Zeta potential is commonly employed to quantitate the magnitude of charge of materials; such charge is an important indicator of the stability and degree of electrostatic repulsion of colloidal dispersions [31, 32]. Myricetin significantly decreased the zeta potential of SEVI fibrils in a dose-dependent manner (Fig. 7d), indicating that it neutralized their surface positive charge.

### Myricetin antagonizes the infection-enhancing properties of human semen

**Fig. 8:**
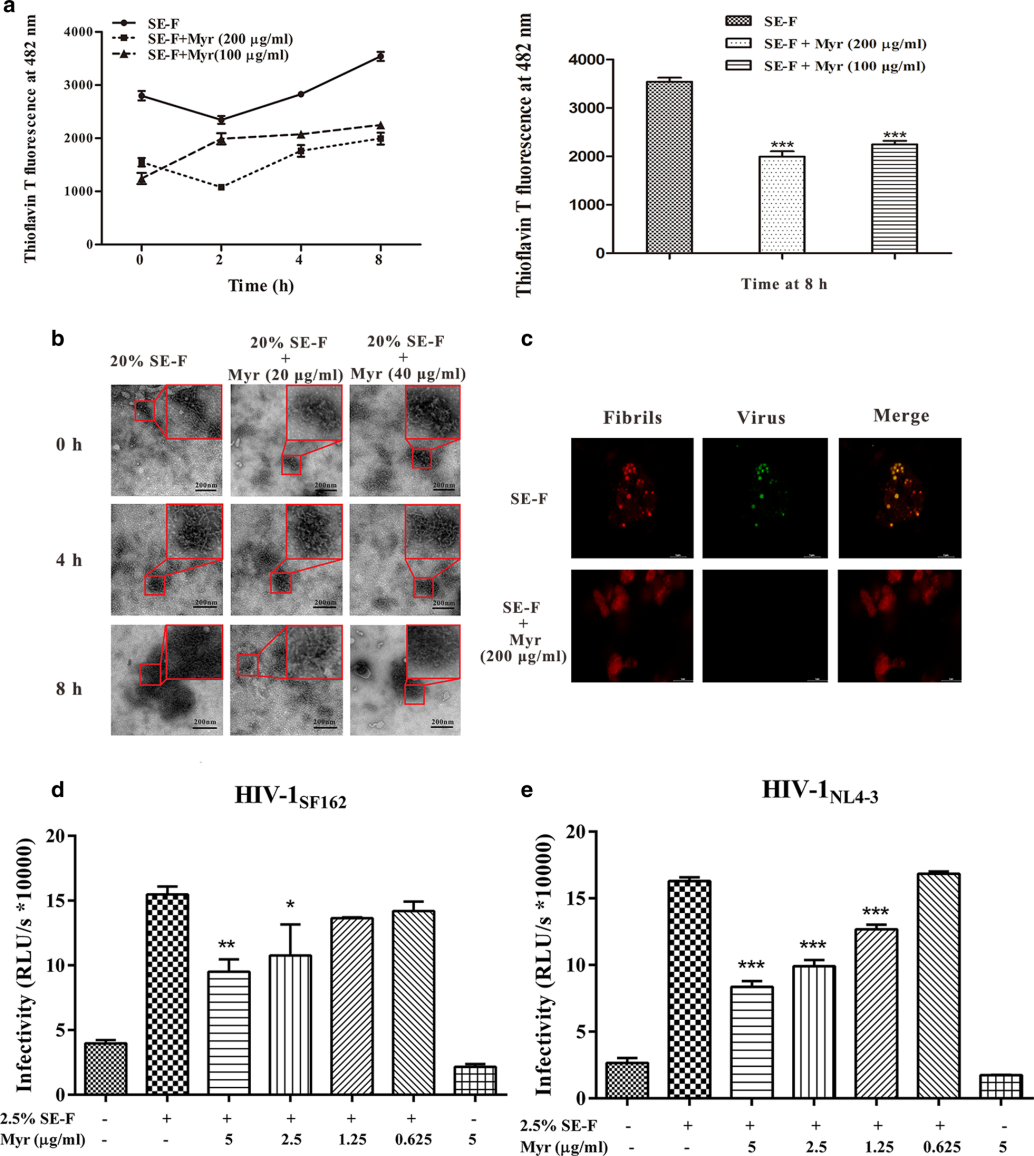
Myricetin antagonizes the infection-enhancing properties of human seminal fluid (SE-F). **a** Seminal amyloid fibril formation was antagonized by myricetin in a dose-dependent manner. SE-F samples containing myricetin (200, 100 or 0 μg/ml) were agitated at 1400 rpm at 37 °C for 8 h. Fibril integrity at the indicated time points (0, 4 and 8 h) was assessed using ThT fluorescence. **b** Seminal fibril samples (1:5 dilution) in the presence or absence of myricetin (20 and 40 μg/ml) at various time points (0, 4 and 8 h), as visualized by TEM. The scale bar is 200 nm. **c** Myricetin inhibited the assembly and attachment of HIV-1 virions to seminal fibrils by fluorescence confocal microscopy. The scale bar is 5 µm. The infection-enhancing properties of SE-F on HIV-1_SF162_ (**d**) and HIV-1_NL4-3_ (**e**) infection were attenuated by myricetin. SE-F samples were incubated with myricetin at various concentrations (200, 100, 50, 25 and 0 μg/ml) for 1 h. After centrifugation, the pellets were dissolved in fresh medium at a final dilution of 1:40 and incubated with various HIV-1 infectious clones. Luciferase activities were measured at 72 h post-infection. Average values (± SD) were calculated from triplicate measurements; the data shown represent one representative trial of three independent experiments. One-way ANOVA with Dunnett’s post hoc multiple comparisons test was used to statistically analyze differences between samples containing SE-F alone and samples containing SE-F and myricetin (*p < 0.05; **p < 0.01, ***p < 0.001)

Multiple studies have reported that semen increases HIV-1 infection in vitro and that semen-mediated promotion of HIV-1 infection correlates with the levels of amyloidogenic fragments in semen. The combined effects of myricetin on fibril architecture and the formation of fibril–virion complexes led us to investigate whether myricetin possibly diminishes the infection-enhancing properties of the amyloid fibrils present in human semen. Figure 8a shows that after agitation in the absence of myricetin, seminal fluid (SE-F) samples displayed a slight increase in fluorescence intensity, indicating the formation of new seminal fibrils. Remarkably, myricetin slightly abrogated the new formation of seminal fibrils in SE-F. To determine whether myricetin depletes or affects semen of endogenous amyloids, we further measured the effects of myricetin using a sensitive TEM assay, the results of which showed weak inhibition of seminal fibril formation by myricetin at a final concentration of 40 μg/ml after agitation of 8 h (Fig. 8b). We further investigated whether myricetin affects the role of endogenous seminal fibrils in enriching virions. Based on fluorescence confocal microscopy, myricetin markedly inhibited the capture of HIV-1 virions by endogenous seminal fibrils (Fig. 8c). In our HIV-1 infection assay, semen was first incubated with myricetin for 1 h, and the sample was then centrifuged to remove the remaining free myricetin to avoid any direct activity against HIV. The treatment of cells with 2.5% SE-F in the absence of myricetin enhanced HIV-1 infection by 15- to 16-fold. In contrast, seminal fibrils alone promoted aggregation of virions and fibril–virion complex formation. However, the addition of myricetin decreased the enhancement of infection by SE-F in a dose-dependent manner, suggesting that myricetin antagonizes semen-mediated infection enhancement by HIV-1_SF162_ (Fig. 8d) and HIV-1_NL4-3_ (Fig. 8e).

### Myricetin shows low cytotoxicity in vitro

**Fig. 9:**
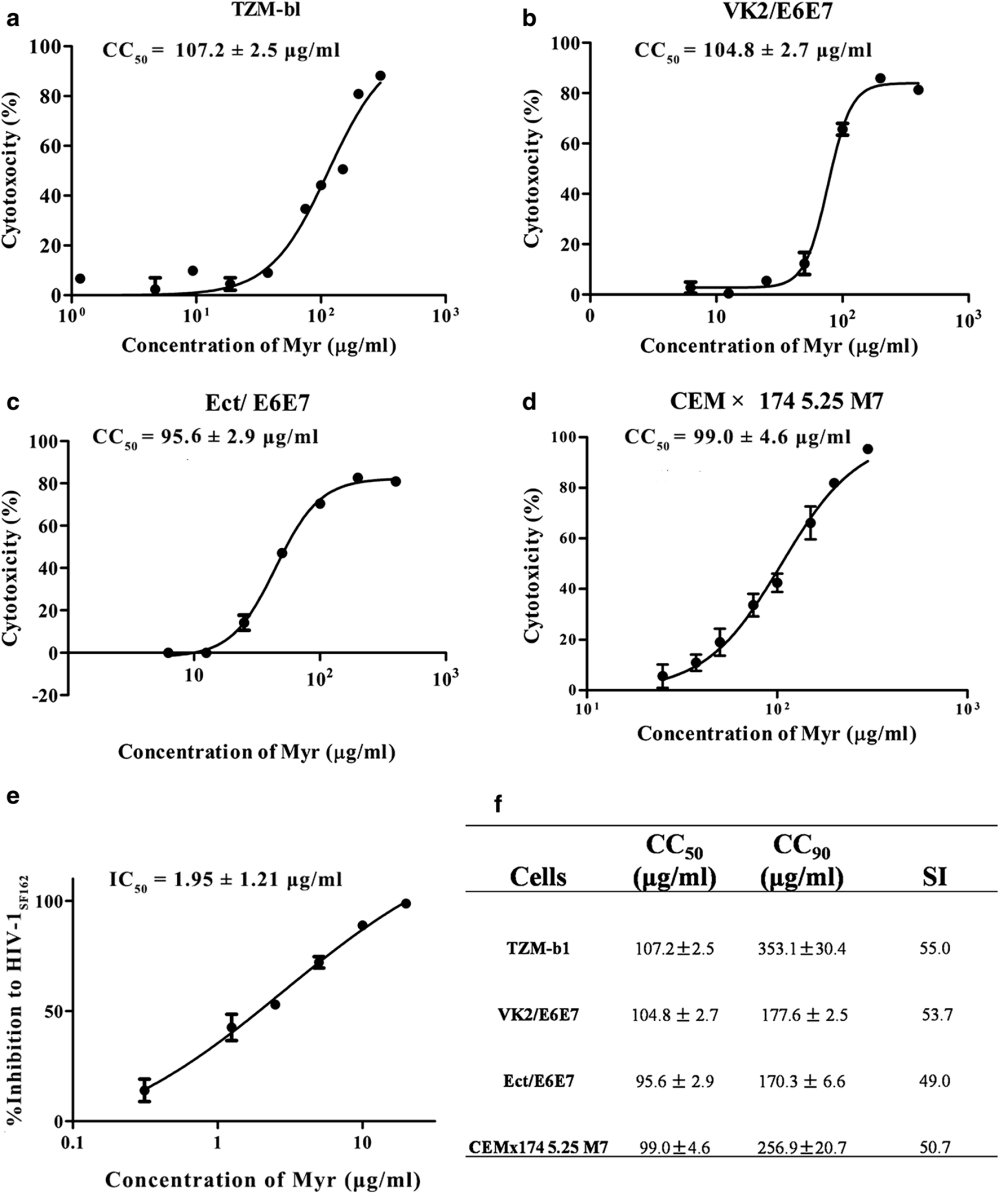
Cytotoxicity of myricetin in vitro. **a** TZM-b1, **b** VK2/E6E7, **c** Ect/E6E7 and **d** CEMx174 5.25 M7 cells. The concentrations of myricetin for (**a**) and (**d**) were 300, 200, 150, 100, 75, 50, 37.5, and 25 μg/ml, for (**b**, **c**) were double dilution from 400 to 6.25 μg/ml. The MTT assay was used to evaluate adherent cells, and the XTT assay was used for cells in suspension. **e** %Inhibition of HIV-1_SF162_ infection by myricetin alone without SEVI. The indicated concentrations of myricetin were incubated with the HIV-1_SF162_ infectious clone for 30 min. The mixtures were then added to prepared TZM-b1 cells. Luciferase activities were measured at 72 h post-infection. Each sample was tested in triplicate, and the data are presented as the mean ± SD. **f** CC_50_, CC_90_ and SI values of myricetin against all tested cells. The assay was performed in triplicate; the data are presented as the mean ± SD

The potential cytotoxic effects of myricetin on HIV target cells (TZM-bl and CEMx174 5.25 M7 cells) and reproductive tract epithelial cells (VK2/E6E7 and Ect/E6E7 cells) were assessed using MTT (3-[4,5-dimethyl-2-thiazolyl]-2,5-diphenyl-2*H*-tetrazolium bromide) or XTT [2,3-bis(2-methoxy-4-nitro-5-sulfophenyl)-5-(phenylamino)carbonyl-2*H*-tetrazolium hydroxide] and tris (2,2-bipyridyl)dichlororuthenium(II)(Ru(bpy)_3_^2+^) assays. Myricetin displayed low cytotoxicity in vitro toward all cell lines tested, with 50% cytotoxic concentration (CC_50_) values ranging from 95.6 to 107.2 μg/ml (Fig. 9a–d). It should be noted that the final concentrations of myricetin used in these experiments were far below the CC_50_ values. At a concentration of 50 µg/ml, myricetin displayed no obvious cytotoxicity toward CEMx174 5.25 M7 or TZM-b1 cells (Fig. 6d). We also assessed direct inhibition of HIV-1_SF162_ infection by myricetin alone and obtained an IC_50_ of 1.952 μg/ml (Fig. 9e). The selectivity index (SI = CC_50_/IC_50_) ranged from 49 to 55, indicating that myricetin might be safe for use in patients (Fig. 9f).

### Combinations of myricetin and ARV drugs in 1% SE-F display synergistic effects against HIV-1 infection

**Fig. 10:**
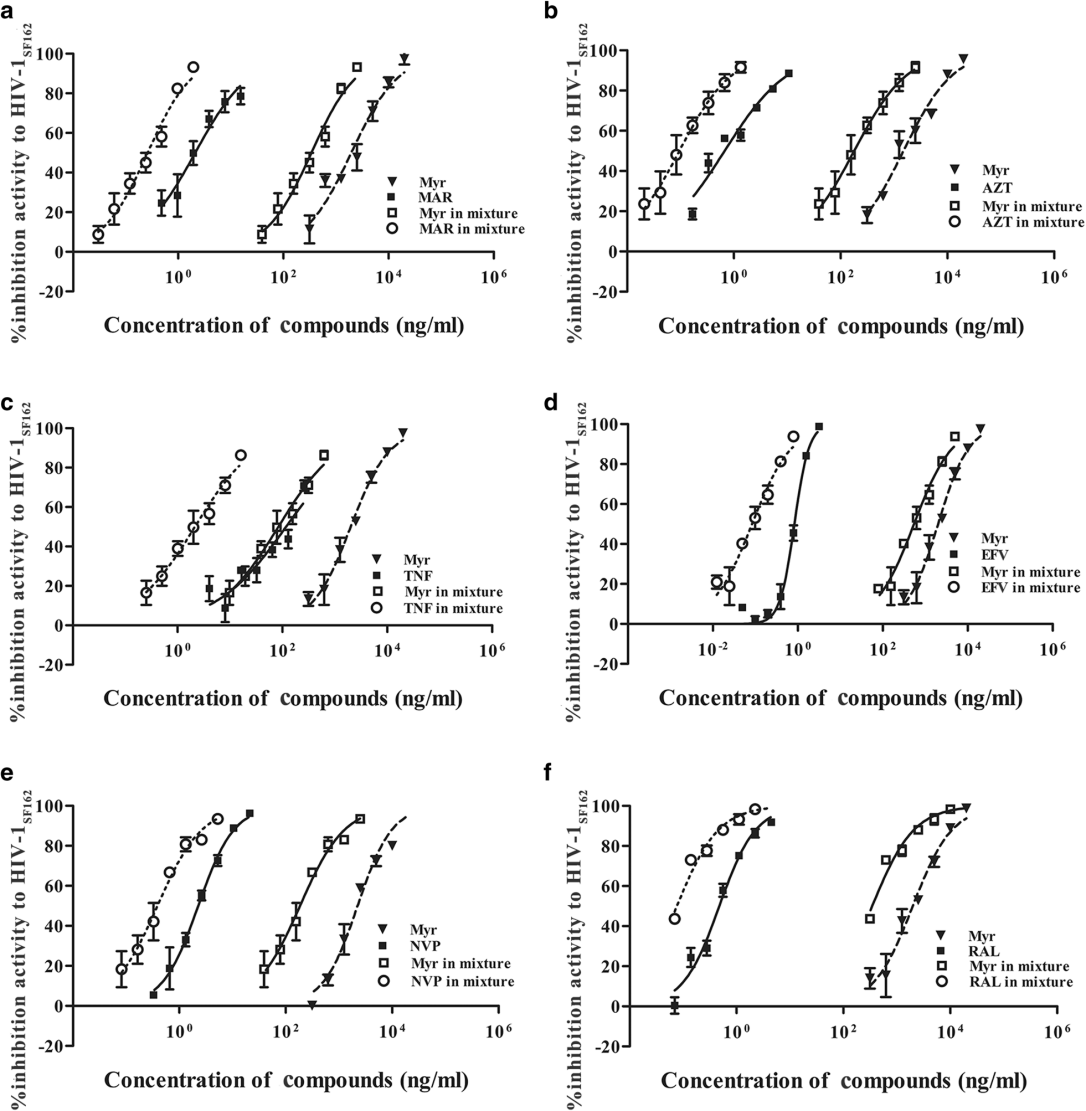
Synergism achieved by combining myricetin with ARVs for inhibition of infection by HIV-1_SF162_ in semen. Compounds were examined at fixed molar ratios individually and in combination. **a** Myr and MAR (2095:1); **b** Myr and AZT (1571:1); **c** Myr and TNF (62.8:1); **d** Myr and EFV (3142:1); **e** Myr and NVP (628:1); **f** Myr and RAL (3142:1). Luciferase activities were measured at 72 h post-infection. Each sample was tested in triplicate, and the data are presented as the mean ± SD

In addition to binding to seminal amyloid fibrils, myricetin also appears to directly inhibit HIV-1 infection by targeting HIV integrase [18]. Accordingly, we determined the overall complementary effects of myricetin combined with various anti-retroviral (ARV) drugs on transmission of HIV-1 through semen, which is the most common route. The capacity of myricetin and ARV drugs alone or in combination to prevent infection of TZM-bl cells by 100 TCID_50_ of HIV-1_SF162_ in 1% SE-F was determined using luciferase assays. The ARV drugs used included an HIV entry inhibitor (maraviroc, MAR), nucleoside reverse transcriptase inhibitors (NRTIs) (zidovudine (AZT) and tenofovir (TNF)), non-nucleoside reverse transcriptase inhibitors (NNRTIs) (nevirapine (NVP) and efavirenz (EFV)) and an integrase inhibitor (raltegravir, RAL). The data in Table 1 show that combining myricetin with any of the above ARV drugs produced strong synergistic and complementary effects against infection by HIV-1_SF162_ in 1% SE-F. The observed 50% competitive index (CI_50_) values ranged from 0.051 to 0.450, and the IC_50_ values of the individual drugs in each combination were reduced by approximately 2.91- to 154.87-fold. The synergistic effects of these combinations were also assessed intuitively, as shown in Fig. 10. The strongest synergism was observed when myricetin was combined with the NRTI TNF: CI_50_ = 0.051, and dose reductions in the IC_50_ values of myricetin and TNF of approximately 24- and 107-fold, respectively, were observed (Table 1, Fig. 10). This combination is expected to exert synergistic antiviral effects and to help prevent the development of drug resistance during the prevention and treatment of HIV infection.

**Table 1:** Combination index (CI) and dose-reduction values for inhibition of HIV-1_SF162_ infection determined by combining myricetin with ARVs in semen^a^

Drug combination, % inhibition	CI^b^	Mean value for^a^:					
		Myr			ARVs		
		Conc. (ng/ml)		Dose reduction	Conc. (ng/ml)		Dose reduction
Conc. (molar ratio)		Alone	Mixture		Alone	Mixture	
Myr:AZT (1571:1)							
50	0.331	600.52	105.97	5.67	0.37	0.06	6.17
90	0.145	16,962.76	1626.10	10.43	17.81	0.87	20.47
Myr:EFV (3142:1)							
50	0.450	2040.81	654.58	3.12	0.80	0.10	8.00
90	0.466	9407.22	2299.93	4.09	1.64	0.36	4.56
Myr:MAR (2095:1)							
50	0.275	1916.80	298.25	6.43	1.92	0.23	8.35
90	0.234	14,712.25	2335.84	6.30	24.03	1.80	13.35
Myr:RAL (3142:1)							
50	0.413	1613.13	372.91	4.33	0.46	0.08	5.75
90	0.514	7554.10	2592.53	2.91	3.37	0.58	5.81
Myr:TNF (62.8:1)							
50	0.051	1976.91	82.31	24.02	113.10	1.06	106.70
90	0.151	9440.37	1366.11	6.91	2721.14	17.57	154.87
Myr:NVP (628:1)							
50	0.262	2199.95	183.01	12.02	2.15	0.38	5.66
90	0.437	15,403.41	1709.88	9.01	11.02	3.59	3.07

^a^The data shown are the means of three independent assays performed in triplicate

^b^The CI value reflects the nature of the interaction between compounds

## Discussion

There is evidence to support that human semen within the context of HIV-1 sexual transmission may have contributed to the failure of microbicides in previous clinical trials [33, 34]. Indeed, the antiviral efficacy of many candidate microbicides is greatly diminished in the presence of semen, and seminal amyloid fibrils have been found to be responsible for the semen-mediated enhancement of HIV infection [4, 9]. Due to their powerful positive charge, amyloid fibrils in semen can effectively capture virus and promote attachment to target cells by neutralizing the inherent electrostatic repulsion between the negative charges on the surfaces of HIV virions and target cells. Thus, a strategy of neutralizing the enhancing activity of seminal amyloid fibrils might be an attractive option for the development of ideal microbicides.

Several agents that block the enhancing activity of seminal amyloid fibrils in HIV infection have been reported. For example, based on its ability to abrogate SEVI-mediated enhancement of HIV-1 infection, the natural ingredient of green tea EGCG appears to be a promising supplement to antiretroviral microbicides [12, 13]. A component of EGCG, gallic acid, is able to directly abrogate the viral enhancement of seminal fibrils and might be able to prevent HIV infection via sexual transmission [35]. Unfortunately, EGCG has poor solubility and poor oral bioavailability and easily undergoes oxidation in vivo, complicating its use in clinical trials [36, 37]. Furthermore, EGCG was recently reported to promote the formation of toxic tau oligomers [38]. The small “molecular tweezer” CLR01 interacts with lysine and arginine residues on SEVI, leading to inhibition of the formation of infectivity-enhancing seminal amyloids and preformed fibril remodeling [29]. Of note, CLR01 interacts promiscuously and nonspecifically with the virus envelope; thus, it is also a broad-spectrum inhibitor of infection by enveloped viruses, including HIV-1, Herpes, Ebola and Zika [39]. The simplest and most logical explanation for the broad range of activity of CLR01 is that the inherent “stickiness” of the molecule confers its promiscuous nature. However, the nonspecific binding of CLR01 to alkaline amino acids in the human body decreases the suitability of CLR01 as a therapy. Therefore, the development of effective, affordable and specific semen fibril antagonists is urgently needed.

The available literature verifies that myricetin possesses the potential to inhibit aggregation of the amyloid fibrils that play an important role in disease. In this work, we explored the potential impact of myricetin on the aggregation of seminal amyloid fibrils. First, myricetin at a concentration of 75 μg/ml was found to completely inhibit amyloidogenesis by peptide PAP248–286 in vitro (Fig. 1a, b); myricetin at a concentration of 200 or 40 μg/ml partially inhibited the formation of new amyloid fibrils in semen, as based on ThT and TEM assays (Fig. 8a, b). The ability of seminal fibrils to cause virion enrichment was inhibited by myricetin at a final concentration of 200 μg/ml (Fig. 8c). Second, myricetin was shown to be capable of blocking the enhancement of viral infection by abrogating interaction between SEVI and HIV-1 virions (Figs. 6, 7). It was clearly demonstrated that an SE-F sample agitated at 37 °C for 8 h significantly lost HIV-enhancing activity compared to the SE-F sample with no agitation [11, 40]. Correspondingly, the ability of semen to enhance HIV-1 infection after the addition of myricetin for 1 h incubation was decreased in a dose-dependent manner (Fig. 8d, e).

It is noteworthy that myricetin is known as a strong inhibitor of HIV reverse transcriptase and integrase, and it is possible that all of the inhibition described above is simply due to the known ability of myricetin to inhibit HIV infection, as opposed to its effects on SEVI. Indeed, we plan to compare the inhibitory activities of myricetin on HIV infection in the absence and presence of SEVI and semen. Regardless, one issue with such a comparison is that SEVI- or semen-mediated enhancement of HIV-1 infection occurs only at low infectivity [5], with myricetin completely preventing HIV infection, even very low concentrations. To avoid the direct activity of myricetin against HIV as much as possible, the mixture of SEVI/semen and myricetin was centrifuged at 12,000 rpm for 5 min to remove free myricetin from the samples. Thus, most of the inhibition caused by myricetin in our study is likely to have resulted from its effects on SEVI. Our findings indicate that myricetin is worth investigating as a possible candidate for the development of an ideal bifunctional microbicide with both anti-HIV and anti-SEVI activities.

According to native acid gel electrophoresis and SPR analyses, myricetin at 12.5 and 6.36 μg/ml was found to be able to bind to the PAP248–286 monomer (Fig. 3a, b). By using a computational molecular docking assay, we found that myricetin binds to specific amino acids in the PAP248–286 monomer, namely, Leu-258, Gln-259, Val-264, Leu-268, Met-271 and Arg-273, through salt bridges, hydrophobic interactions, and hydrogen bonds (Fig. 3e). Van der Waals forces may contribute to the interaction between myricetin and PAP248–286. Interestingly, the six residues identified as being involved in myricetin and PAP248–286 interaction are contained within the reported functional regions of PAP248–286. PAP257–267 is considered to be the central region of PAP248–286, and this amyloidogenic region of the SEVI precursor peptide PAP248–286 has high fibril-forming propensity. Coincidentally, PAP268–271 has been reported to play an important role in promoting SEVI fibrillation [23]. Residues G260–N265 are thought to be involved in the initiation of fibrillation [40], and residues V262–H270 were shown to be the amyloidogenic region for SEVI fibrillation [41]. Therefore, one or several of the residues present at those six sites might be essential for the formation of SEVI fibrils [42]. The results of our study confirmed that wild-type PAP257–273, as well as PAP257–273 mutants containing Leu258A, GIn259A, Leu268A and Arg273A, readily formed amyloid fibrils (Fig. 3f). Notably, Val264A and Met271A mutations of PAP257–273 exhibited attenuated aggregation during fibril formation, indicating that the Val264 and Met271 residues of PAP248–286 may play a critical role in the assembly of PAP248–286 into fibrils. Myricetin clearly inhibited fibril formation by wild-type PAP257–273 and by four of the PAP257–273 mutants in a dose-dependent manner (Fig. 3g). These results suggest that inhibition of SEVI fibril formation by myricetin might be caused by the binding of myricetin to Val264 and Met271 of PAP248–286.

It is generally recognized that the formation of mature amyloid fibrils involves three stages: (i) monomers are first assembled into oligomers; (ii) fibrils evolve from protofibrils after nucleation; and (iii) the fibrils rapidly aggregate until equilibrium is reached [43]. Continuing investigations suggest that an oligomeric form of amyloid proteins plays a key role in disease causation and that low-*n*-order oligomers are especially important [28]. It would therefore be expected that the most efficacious therapeutic agents would be those that block the early assembly processes associated with amyloid protein oligomerization. Using a PICUP assay, we found that myricetin efficiently abrogated oligomerization of PAP248–286 in a dose-dependent manner (Fig. 4a). CD data also confirmed that myricetin at 5 μg/ml successfully inhibited the formation of β-sheet oligomers by PAP248–286 monomers (Fig. 1e).

Notably, myricetin decreased the zeta potential of SEVI in a dose-dependent manner (Fig. 7d). This might reflect a completely independent mechanism of inhibition of SEVI activity that is distinct from fibrillation inhibition. In general, the molecular electron-donating ability of a compound is determined by the highest occupied molecular orbital (HOMO) eigenvalue. Myricetin is a highly negatively charged compound with a HOMO eigenvalue of − 5.7 eV [44]. The electrostatic interaction between myricetin and SEVI fibrils might cause myricetin to shield the surface cationic property of SEVI fibrils, leading to competitive inhibition of the binding of SEVI to the virus. In a similar manner, virus pull-down assays confirmed that myricetin inhibits the binding of HIV to SEVI fibrils in a dose-dependent manner (Fig. 7a–c). As shown in Additional file 1: Figure S4, myricetin did not affect the total amount of viral core protein p24, an observation that tends to exclude an antiviral effect of myricetin on the virus particles themselves. The observations are consistent with the idea that myricetin is an integrase inhibitor that affects the stage of viral growth but has no effect on the viral envelope.

Although anionic polymers are effective inhibitors of SEVI activity and HIV infection, they are not ideal candidates as anti-HIV vaginal microbicides because they are highly pro-inflammatory, disrupting the vaginal epithelial barrier [45]. One question that arises is does intravaginal application of anionic myricetin cause inflammation in female genital tract? There is a growing body of data indicating that myricetin displays strong anti-inflammatory activity in a variety of in vitro assays, as well as in both acute and chronic in vivo animal models, through regulation of multiple signaling pathways [15, 46, 47]. Therefore, myricetin with anti-inflammatory, anti-HIV and anti-SEVI activities might be developed as a promising microbicide to reduce the risk of HIV infection.

Previous studies have reported that fresh ejaculate contains semen fibrils; thus, assembly blockers might be inactive [10]. However, it is rare that agents exhibit the ability to degrade highly stable amyloid fibrils. Fortunately, we found that myricetin degraded SEVI fibrils in a dose-dependent manner, resulting in a lack of ability to seed the assembly of soluble PAP248–286 by SEVI (Fig. 5a, b). In addition, CD results showed that myricetin could immediately remodel SEVI into non-templating conformers (Fig. 5c). One of the advantages of using myricetin is that degradation occurs immediately, whereas other reported remodeling inhibitors, including EGCG and CLR01, must be applied after several hours [12, 29]. Another advantage of using myricetin is its broad inhibition of the formation of seminal fibrils including SEVI, SEM1 and SEM2. Consequently, it is preferable to develop semen fibril antagonists that not only antagonize fibrillation but also remodel preformed fibrils. Future studies designed to determine the exact mechanism of action of myricetin with regard to the degradation of fibrils should be conducted.

It has previously been reported that brief exposure of HIV to semen reduced the antiviral efficacy of most microbicides [48]. Notably, myricetin is known to be an inhibitor of HIV-1 integrase, and we found that myricetin can partially weaken the semen-mediated enhancement of viral infection. Moreover, myricetin displays synergistic effects against HIV-1 infection in combination with other ARV agents in semen (Table 1; Fig. 10). As a key ingredient of various human foods and beverages, myricetin is relatively safe when used in patients. Our results also confirmed that myricetin has low cytotoxicity toward all tested cell lines in vitro, with CC_50_ values ranging from 95.6 to 107.2 μg/ml (Fig. 9). Therefore, it is likely that myricetin can be further developed into a candidate for inclusion in a combination microbicide to prevent HIV-1 sexual transmission.

Increasing evidence indicates that endogenous amyloids play natural and biological roles in the human body [49]. It was recently reported that semen amyloids may play a role in reproduction by participating in sperm selection and facilitating spermatozoal clearance [50]. Positively charged SEVI fibrils exert indirect antimicrobial activity by trapping microbial pathogens [51], though it remains unclear how inhibitors of amyloid formation or action may affect the antimicrobial and sperm-clearing natural role of amyloids in semen. To date, no studies have reported the side effects of semen fibril antagonists on the normal physiological function of amyloids. Before a semen fibril-targeted agent is clinically available, its potential effects on the normal physiological function of amyloids should be tested.

## Conclusions

In this study, we found that myricetin interferes with various essential steps in the formation and remodeling of SEVI fibrils. Myricetin significantly inhibited all stages of SEVI formation, including early oligomerization and fiber extension, thereby blocking SEVI-mediated enhancement of HIV-1 infection. In addition, myricetin is capable of remodeling seminal amyloid fibrils and antagonizing semen-mediated enhancement of HIV-1 infection. Notably, myricetin displays synergistic effects against HIV-1 infection in semen when combined with other ARV agents and may work synergistically to reduce the rate of HIV transmission. It is likely that myricetin can be developed into a dual-functional microbicide candidate with both anti-HIV-1 and anti-SEVI-enhancing activities for use in preventing sexual transmission of HIV-1.

## Methods

### Cell lines, plasmids and reagents

TZM-bl, VK2/E6E7, Ect/E6E7 cells were obtained from the National Institutes of Health AIDS Research and Reference Reagent Program. HEK-293 T cells were purchased from American Type Culture Collection (ATCC) (Manassas, VA, USA). CEMx174 5.25M7 cells were kindly provided by Dr. C. Cheng-Mayer. Plasmids encoding CXCR4-tropic HIV-1_NL4-3_ and CCR5-tropic HIV-1_SF162_ infectious clones were kindly provided by Jan Münch of Ulm University (Ulm, Baden-Württemberg, Germany). The peptides PAP248–286, SEM1_86–107_, SEM2_86–107_ and N36 were synthesized by Scilight Biotechnology LLC (Beijing, China). Myricetin was purchased from Chengdu Must Biotechnology Co., Ltd. (Chengdu, China). ProteoStat Amyloid Plaque Detection Kits were purchased from Enzo Life Sciences (Plymouth Meeting, PA, USA). Thioflavin T (ThT), phlorizin, GST, Congo red, ammonium persulfate, DL-dithiothreitol (DTT), MTT, and XTT were purchased from Sigma (St. Louis, MO, USA). An anti-p24 monoclonal antibody (183-12H-5C), anti-HIV IgG, zidovudine (AZT), raltegravir (RAL) and maraviroc (MAR) were obtained from the National Institutes of Health AIDS Research and Reference Reagent Program. Tenofovir (TNF), nevirapine (NVP) and efavirenz (EFV) were purchased from Promega Biotech Co., Ltd. (Madison, WI, USA). Rabbit anti-PAP IgG was prepared, produced and purified by AbMax Biotechnology (Beijing, China).

### Semen and seminal fluid samples

Pooled semen samples were obtained from 20 healthy donors at Nanfang Hospital (Guangzhou, China) after they had provided informed consent to participate in the study. Seminal fluid (SE-F) samples were collected by centrifugation of semen samples for 15 min at 10,000 rpm at 4 °C to remove spermatozoa and debris and were then stored at − 20 °C. The research protocols for this study were approved by the Ethical Committee of Nanfang Hospital of Southern Medical University and performed in accordance with relevant guidelines and regulations.

### Formation of fibrils by PAP248–286, SEM1_86–107_, SEM2_86–107_ and formation of fibrils in semen

Solutions of the peptides PAP248–286, SEM1_86–107_ and SEM2_86–107_ (3 mg/ml) were agitated in the presence or absence of myricetin at the indicated concentrations (75, 37.5, 18.75 and 9.375 μg/ml for SEVI or 200, 100, 50 and 25 μg/ml for SEM1/2) for 48 h at 1400 rpm at 37 °C using an Eppendorf Thermomixer (Hamburg, Germany). At the indicated time points (0, 6, 12, 18, 24 and 48 h), aggregates were collected and stored at − 20 °C.

SE-F samples were collected and agitated at 1400 rpm at 37 °C in the presence or absence of myricetin at the indicated concentrations (200 and 100 μg/ml) for 8 h. At the indicated time points (0, 2, 4 and 8 h), aggregates were collected and stored at − 20 °C.

### Remodeling of SEVI fibrils

SEVI fibrils (3 mg/ml) were agitated in the presence of myricetin (500, 250, 125 and 0 μg/ml) for 3 days at 37 °C at 1400 rpm. At various time points (1 min, 12, 24, 36, 48, 60 and 72 h), the sample mixtures were tested in ThT, Congo red and CD assays. Myricetin alone (500 μg/ml) was used as a negative control.

### ThT fluorescence assay

Ten microliters of sample prepared as described above was mixed with 195 μl of ThT working solution (50 μM). The fluorescence of the mixture was measured using an RF-5301 PC spectrofluorophotometer (Shimadzu) at an excitation wavelength of 440 nm and an emission wavelength of 482 nm [23, 52].

### Congo red staining assay

Five microliters of sample were mixed with 200 μl of Congo red reagent obtained from a Congo red kit for 2 min at room temperature (RT). The samples were then centrifuged at 12,000 rpm for 5 min, and the red-colored fiber precipitate was dissolved in 50 μl of dimethyl sulfoxide (DMSO). The absorbance of the solution at 490 nm was recorded using an enzyme-linked immunosorbent assay (ELISA) reader (Tecan, Research Triangle Park, NC, USA) [23, 52].

### TEM

Suspensions of the collected samples (the final concentration of PAP248–286 was 500 μg/ml, and the final dilution of SE-F was 1:5) were deposited onto glow-discharged, carbon-coated grids for 2 min. The grids were then negatively stained with 2% phosphotungstic acid for an additional 2 min. The morphology of SEVI or seminal fibrils in the presence and absence of myricetin (16, 8 μg/ml for SEVI or 40, 20 μg/ml for semen) was visualized using an H-7650 TEM (Hitachi Limited, Tokyo, Japan). Myricetin (16 μg/ml) was used as a negative control.

### CD

The secondary β-sheet structure of SEVI fibrils (the final concentration of PAP248–286 in the sample was 300 μg/ml) in the presence and absence of myricetin (5 μg/ml of the formation or 50, 12.5 μg/ml of the remodeling) was determined by CD spectroscopy in the “far-UV” spectral region (198–260 nm) using a Jasco 715 spectropolarimeter equipped with a thermostat-controlled cell housing and cells with a 1-mm path length (Jasco Inc., Japan). Each CD spectrum was collected three times [53].

### HIV-1 infection assay

Enhancement of SEVI fibrils and SE-F samples in the presence and absence of myricetin was measured using HIV-1 infection assays as previously described [30, 54]. First, SE-F samples were collected as described above. Next, the SE-F samples were incubated with myricetin at various concentrations (200, 100, 50, 25 and 0 μg/ml) for 1 h at 37 °C; SEVI fibrils (50 μg/ml) were first incubated with graded concentrations of myricetin (50, 25, 12.5, 6.25, 3.13, 1.56, 0.78, 0.39 and 0 μg/ml) for 30 min. To eliminate the anti-HIV activity of myricetin itself as much as possible, the SEVI or SE-F and myricetin mixtures were centrifuged at 12,000 rpm for 5 min to remove free myricetin. The pellets of SE-F samples were dissolved in fresh medium at a final dilution of 1:40. Thus, the final concentrations of myricetin in 2.5% SE-F were 5, 2.5, 1.25, 0.625 and 0 μg/ml. The pellets of SE-F and SEVI fibrils in fresh medium were then incubated with CXCR4-tropic HIV-1_NL4-3_ and CCR5-tropic HIV-1_SF162_ infectious clones at RT for 10 min. Subsequently, 100 μl of the fibril–virus mixture was added to TZM-bl cells, which were cultured at 37 °C for 3 h, after which the culture supernatant was replaced with fresh medium. At 3 days post-infection, the cells were collected, washed and lysed with lysing reagent, and the luciferase activity was measured using a luciferase assay kit (Promega, Corp., Madison, WI, USA). HIV and myricetin were used as negative controls. The viability of TZM-bl cells with or without SEVI fibrils was detected in parallel with all infection assays under the exact same conditions.

The direct anti-HIV activity of myricetin on HIV-1_SF162_ infection was detected. First, 1 × 10^5^/ml TZM-bl cells were seeded in 96-well plates at 37 °C overnight, and 100 TCID_50_ HIV-1 virus was incubated with myricetin (20, 10, 5, 2.5, 1.25, 0.625, 0.3125 and 0 μg/ml) at graded concentrations at 37 °C for 30 min. The mixture was then added to cells and further incubated for 72 h. The cells were collected, washed and lysed with the lysing reagent included in a luciferase assay kit. IC_50_ values were calculated using CalcuSyn software [55], kindly provided by T. C. Chou (Sloan-Kettering Cancer Center, New York, NY).

### Cytotoxicity assay in vitro

The cytotoxicity of myricetin toward TZM-bl, VK2/E6E7 and Ect/E6E7 cells was evaluated using MTT assays, and the cytotoxicity of myricetin toward CEMx174 5.25 M7 cells was evaluated using an XTT assay. Briefly, approximately 90% confluent cells were plated in 96-well plates at 1 × 10^5^/ml, and the plates were incubated at 37 °C overnight. Different concentrations of myricetin were added, and the cells were incubated for an additional 48 h at 37 °C. For the MTT assays, the culture supernatant was discarded, and 100 μl of 0.5 mg/ml MTT solution was added to the cells. After incubating the cells for an additional 4 h, the cell-free supernatant was removed, and the formazan crystals formed were dissolved in 150 μl of DMSO. The absorbance of the resulting solution at 570 or 450 nm was measured using an ELISA reader. For the XTT assay, 50 µl of XTT solution (1 mg/ml) containing 0.02 µM phenazine methosulfate (PMS) was added. After 4 h, the absorbance at 450 nm was measured using an ELISA reader. CC_50_ was calculated using CalcuSyn software.

### Fluorescence microscopy and flow cytometry assays

Lymphoid CEMx174 5.25M7 cells that had been stably transduced with a long terminal repeat (LTR)-luciferase and LTR-green fluorescent protein cassette were used as target cells [5]. SEVI (100 μg/ml) was incubated with myricetin (50 μg/ml) at 37 °C for 30 min; the samples were then centrifuged at 12,000 rpm for 5 min to remove free myricetin. The resulting pellets were dissolved in fresh medium and mixed with an equal volume of HIV-1_SF162_ for 5 min at RT. The mixture was then added to CEMx174 5.25M7 cells, which were cultured at 37 °C for 48 h. The fluorescence intensity of the resulting cell suspensions was observed by fluorescence microscopy (Nikon, Japan). The cells were washed, resuspended and analyzed by flow cytometry (BD FACSCanto TM^Π^). HIV and myricetin were used as negative controls. The viability of CEMx174 5.25M7 cells with or without SEVI fibrils was detected in parallel with all infection assays under the exact same conditions.

### Seeding function of preformed SEVI

Preformed SEVI seeds (200 μg/ml) were added to 2 mg/ml PAP248–286 peptide solution in the presence or absence of myricetin (500, 200, 100, 80, 40, 20, 10 and 1 μg/ml), and the mixture was agitated at 1400 rpm at 37 °C. At the indicated time points (0, 2, 4, 6, 8, 10, and 12 h), samples were collected and subjected to ThT fluorescence assays as described above [29]. The IC_50_ values of myricetin for the formation of PAP248–286 with or without SEVI seeds at 48 h time points were calculated using CalcuSyn software.

### Virus pull-down assays

SEVI fibrils (200 μg/ml) were incubated with myricetin at various concentrations (300, 60, 12, 2.4, 0.48 and 0 μg/ml) at 37 °C for 30 min. The mixture was then challenged with 100 ng/ml HIV-1 virions (HIV-1_NL4-3_ and HIV-1_SF162_) for an additional 30 min. The samples were centrifuged at 12,000 rpm for 5 min, and the supernatants were removed. p24 antigens present in the pellets were detected by ELISA as previously described [56]. Total p24 antigens of the mixtures with myricetin at various concentrations and 100 ng/ml HIV-1 virions (HIV-1_NL4-3_ and HIV-1_SF162_) without SEVI fibrils were detected in parallel using virus pull-down assays under the exact same conditions.

### Confocal microscopy

eGFP-labeled HIV-1 virions (R5-tropic) were produced by transfecting HEK-293T cells with proviral DNA expression plasmids and eGFP-Vpr plasmids by polyethylenimine (PEI) transfection. Semen samples were stained with a Proteostat dye contained in ProteoStat Amyloid Plaque Detection Kit at RT [10]. After staining, the fibril samples were incubated 1:1 with eGFP-labeled HIV-1 virions (100 ng/ml p24) with or without myricetin (200 μg/ml) at 37 °C for 3 h. The samples were then centrifuged at 5000 rpm for 3 min and imaged using a laser scanning Nikon A1 confocal microscope (Nikon, Japan). Proteostat and eGFP were excited using a 561 nm and a 488 nm laser line, respectively, and the emissions were collected using appropriate beam splitters.

### Zeta potential

SEVI (200 μg/ml) was incubated in the presence or absence of myricetin at various concentrations (300, 150 and 30 μg/ml) for 30 min at RT. The samples were then centrifuged at 12,000 rpm for 5 min to remove free myricetin. The resulting pellets were resuspended in 1 mM KCl buffer, and the surface zeta potential of the resuspended samples was measured using a Zeta Nanosizer (Malvern Instruments, UK) [29].

### WB assay

PAP248–286 monomers (100 μg/ml) were incubated with serially diluted myricetin (12.5, 6.25, 3.13 and 1.56 μg/ml) at 37 °C for 30 min, and the samples were centrifuged at 5000 rpm for 15 min. The PAP248–286 peptides remaining in the supernatant were analyzed by electrophoresis through 10% polyacrylamide continuous native gels as previously described [52]. The gels were subjected to WB analysis to specifically identify the remaining PAP248–286 peptides using rabbit anti-PAP IgG.

### Photo-induced cross-linking of unmodified proteins (PICUP)

Samples were chemically cross-linked using PICUP as previously reported [28]. First, PAP248–286 monomer (500 μg/ml) was incubated with various concentrations of myricetin (20, 10, 5 and 2.5 μg/ml) at 37 °C for 30 min. Next, 1 μl of 40 mM tris(2,2-bipyridyl) dichlororuthenium(II)(Ru(bpy)_3_^2+^) and 1 μl of 800 mM ammonium persulfate were added to 18 μl of the mixed sample. The mixture was then exposed to visible light for 5 s, and the cross-linking reaction was terminated by the addition of 2 μl of 5 M DTT. Non-cross-linked PAP248–286 monomer served as a negative control. The samples were separated by 10–20% gradient tricine sodium dodecyl sulfate-polyacrylamide gel electrophoresis (SDS-PAGE), and Coomassie blue staining was used to determine the frequency distribution of monomers and oligomers of PAP248–286. An irrelevant protein (GST) and an irrelevant flavonoid (phlorizin) were used as negative controls.

### Computational docking analysis

The binding of PAP248–286 (RCSB Protein Data Bank (PDB) No. 2L3H) to myricetin was analyzed using the extensible molecular visualization UCSF Chimera program of AutoDock software [42, 57]. High-quality atomic charges for computer simulations of organic molecules in polar media can be generated by a novel charge model called AM1-BCC [58]. Hydrogen atoms were added using the Dock Prep module. The protein–ligand interaction online analysis tool Protein–Ligand Interaction Profiler [59] was applied, and images were generated by PyMOL software [31].

### SPR analysis

The affinity of myricetin binding to PAP248–286 was measured using the BIAcore T100 system (GE Healthcare, Sweden) as previously described [60]. After PAP248–286 (50 µg/ml) was attached to the surface of a CM5 sensor chip as a cross-linker, myricetin (6.36, 3.18, 1.59, 0.80, 0.40 and 0.20 µg/ml) was injected at a flow rate of 20 μl/min, with a contact time of 2 min and a dissociation time of 2.5 min. The running buffer was water. The chip platform was regenerated with 10 mM HEPES, 150 mM NaCl, and 0.01% vol/vol Tween 20 (pH 7.4) and washed with the running buffer. A binding affinity (*K*_*D*_) value was calculated using BIAcore software. An irrelevant peptide (N36) and an irrelevant flavonoid (phlorizin) were used as negative controls.

### Combination treatment in SE-F

The inhibitory activity of myricetin and other ARV drugs and combinations on infection by HIV-1_SF162_ infectious clones was determined as previously described [30, 31, 61]. Briefly, HIV-1_SF162_ at 100 TCID_50_ (50% tissue culture infective dose) was incubated in the presence or absence of various concentrations of myricetin, ARV drugs or combinations thereof in SE-F at 37 °C for 30 min. SE-F was diluted to 1:100 to prevent cytotoxicity, which slightly enhanced HIV infection. The mixture of myricetin, ARV drugs or combinations in 1% SE-F was added to TZM-bl cells, and the cells were incubated for 3 h at 37 °C; the medium was then replaced with fresh medium. Compounds were examined at fixed molar ratios in combination according to their individual IC_50_ values. At 72 h post-infection, the luciferase activity of the cell cultures was measured as described above. The effective concentrations resulting in 50 and 90% inhibition (EC_50_ and EC_90_) were calculated using CalcuSyn software. The data were analyzed for cooperative effects using the Chou-Talalay method. Combination index (CI) values were calculated using the CalcuSyn program. The CI value reflects the nature of the interaction between compounds. CI values of < 1, = 1 and > 1 indicate synergy, additivity and antagonism, respectively. In detail, CI < 0.10 indicates very strong synergism, 0.10–0.30 indicates strong synergism, 0.30–0.70 indicates synergism, 0.70–0.85 indicates moderate synergism, and 0.85–0.90 indicates slight synergism. Dose reductions were calculated as the ratio of the EC_50_ values of the compounds when used alone and when used in combination.

### Statistical analysis

Statistical analysis of the experimental data was performed using a one-way analysis of variance (ANOVA) test in GraphPad Prism 5.0 (San Diego, CA, USA). A *p* value of < 0.05 was regarded as statistically significant; the probability level is indicated by single or multiple asterisks (*) (*p < 0.05; **p < 0.01; ***p < 0.001). All values represent the mean ± SD (standard deviation) of at least three measurements.

## Additional file

**Additional file 1: Figure S1.** Myricetin inhibits other seminal amyloid fibril formation, as shown by ThT assays. (**a**) SEM186-107; (**b**) SEM286-107. Peptide (3 mg/ml) was incubated with myricetin (200, 100, 50 and 10 μg/ml) and agitated at 1,400 rpm at 37 °C. Then samples were collected and monitored by ThT. Average values (± SD) were calculated from triplicate measurements, and the data represent one representative trial of three independent experiment. **Figure S2**. Amyloid fibril samples display loss of enhancement of HIV-1 infection in the presence of myricetin. The raw luciferase activities of mixed SEVI fibril samples prepared in the presence or absence of myricetin with HIV-1_SF162_ (**a**) and HIV-1_NL4-3_ (**b**) infectious clones. The values shown here represent the mean ± SD (n = 3). One-way ANOVA with Dunnett’s post hoc multiple comparisons test was used to statistically analyze the differences between samples containing PAP248-286 alone and samples containing PAP248-286 and myricetin (*p < 0.05; **p < 0.01, ***p < 0.001). **Figure S3**. SEVI (50 μg/ml) was incubated with myricetin at various concentrations (50, 25, 12.5, 6.25, 3.13, 1.56, 0.78 and 0.39 μg/ml). The mixtures were washed one to five times with PBS buffer and centrifuged to remove soluble myricetin. The pellets were resuspended in the original volume of medium and mixed with CCR5-tropic HIV-1_SF162_ (**a**) or CXCR4-tropic HIV-1_NL4-3_ (**b**). The luciferase activities of the cultures were measured at 72 h post-infection. Average values (± SD) were calculated from triplicate measurements; the data shown here represent one representative trial of three independent experiments. One-way ANOVA with Dunnett’s post hoc multiple comparisons test was used to statistically analyze the differences between samples containing SEVI alone and samples containing SEVI and myricetin (*p < 0.05; **p < 0.01, ***p < 0.001). **Figure S4**. The total p24 antigens of the mixtures of myricetin at various concentrations and 100 ng/ml HIV-1 virions without SEVI fibrils were detected in parallel with virus pull-down assays under the exact same conditions. (**a**) HIV-1_SF162_ and (**b**) HIV-1_NL4-3_. Average values (± SD) were calculated from triplicate measurements, and the data represent one representative trial of three independent experiment.
