# Supplementary material for: Myricetin antagonizes semen-derived enhancer of viral infection (SEVI) formation and influences its infection-enhancing activity
Source: Retrovirology. 2018 Jul 16;15:49. doi: 10.1186/s12977-018-0432-3 (PMC6048764; doi:10.1186/s12977-018-0432-3)
Supplement: Supplementary file 1 — Additional file 1: Figure S1. Myricetin inhibits other seminal amyloid fibril formation, as shown by ThT assays. (a) SEM186-107; (b) SEM286-107. Peptide (3 mg/ml) was incubated with myricetin (200, 100, 50 and 10 μg/ml) and agitated at 1,400 rpm at 37 °C. Then samples were collected and monitored by ThT. Average values (± SD) were calculated from triplicate measurements, and the data represent one representative trial of three independent experiment. Figure S2. Amyloid fibril samples display loss of enhancement of HIV-1 infection in the presence of myricetin. The raw luciferase activities of mixed SEVI fibril samples prepared in the presence or absence of myricetin with HIV-1SF162 (a) and HIV-1NL4-3 (b) infectious clones. The values shown here represent the mean ± SD (n = 3). One-way ANOVA with Dunnett’s post hoc multiple comparisons test was used to statistically analyze the differences between samples containing PAP248-286 alone and samples containing PAP248-286 and myricetin (*p < 0.05; **p < 0.01, ***p < 0.001). Figure S3. SEVI (50 μg/ml) was incubated with myricetin at various concentrations (50, 25, 12.5, 6.25, 3.13, 1.56, 0.78 and 0.39 μg/ml). The mixtures were washed one to five times with PBS buffer and centrifuged to remove soluble myricetin. The pellets were resuspended in the original volume of medium and mixed with CCR5-tropic HIV-1SF162 (a) or CXCR4-tropic HIV-1NL4-3 (b). The luciferase activities of the cultures were measured at 72 h post-infection. Average values (± SD) were calculated from triplicate measurements; the data shown here represent one representative trial of three independent experiments. One-way ANOVA with Dunnett’s post hoc multiple comparisons test was used to statistically analyze the differences between samples containing SEVI alone and samples containing SEVI and myricetin (*p < 0.05; **p < 0.01, ***p < 0.001). Figure S4. The total p24 antigens of the mixtures of myricetin at various concentrations and 100 ng/ml HIV-1 virions with [file 12977_2018_432_MOESM1_ESM.doc]

**Myricetin antagonizes semen-derived enhancer of viral infection (SEVI) formation and influences its infection-enhancing activity**

Ruxia Ren^1, 2^**^†^**, Shuwen Yin^1^**^†^**, Baolong Lai^2^, Lingzhen Ma^1^, Jiayong Wen^1^, Xuanxuan Zhang^1^, Fangyuan Lai^1^, Shuwen Liu^1*^, Lin Li^1*^

^1^ Guangdong Provincial Key Laboratory of New Drug Screening, Guangzhou Key Laboratory of Drug Research for Emerging Virus Prevention and Treatment, School of Pharmaceutical Sciences, Southern Medical University, Guangzhou, 510515, China

^2^ Department of Pharmacy, The Seventh Affiliated Hospital of Sun Yat-sen University, Shenzhen, 518107, China

*Corresponding authors. School of Pharmaceutical Sciences, Southern Medical University, 1838 Guangzhou Avenue North, Guangzhou, Guangdong 510515, China
*E-mail addresses:* li75lin@126.com (L. Li), liusw@smu.edu.cn (S. Liu)

*Co-authors’ E-mail addresses:* [Remiren91@126.com](mailto:Remiren91@126.com) (RR), [yinshuwen520@outlook.com](mailto:yinshuwen520@outlook.com) (SY), [laibaolong_cn@126.com](mailto:laibaolong_cn@126.com) (BL), [m15692427949@163.com](mailto:m15692427949@163.com) (LM), [m13528357183@163.com](mailto:m13528357183@163.com ) (JW), [phil2424123@163.com](mailto:phil2424123@163.com) (XZ), [fy13650294644@163.com](mailto:fy13650294644@163.com) (FL), [liusw@smu.edu.cn](mailto:liusw@smu.edu.cn) (SL)*, [li75lin@126.com](mailto:li75lin@126.com) (LL)*

**^†^**These authors contributed equally to this work.

**Supplementary Figure Legends**

**Fig. s1.** Myricetin inhibits other seminal amyloid fibril formation, as shown by ThT assays. (**a**) SEM1_86-107_; (**b**) SEM2_86-107_. Peptide (3 mg/ml) was incubated with myricetin (200, 100, 50 and 10 μg/ml) and agitated at 1,400 rpm at 37°C. Then samples were collected and monitored by ThT. Average values (±SD) were calculated from triplicate measurements, and the data represent one representative trial of three independent experiment.

**Fig. s2.** Amyloid fibril samples display loss of enhancement of HIV-1 infection in the presence of myricetin. The raw luciferase activities of mixed SEVI fibril samples prepared in the presence or absence of myricetin with HIV-1_SF162_ (**a**) and HIV-1_NL4-3_ (**b**) infectious clones. The values shown here represent the mean ± SD (n=3). One-way ANOVA with Dunnett’s post hoc multiple comparisons test was used to statistically analyze the differences between samples containing PAP248-286 alone and samples containing PAP248-286 and myricetin (**p*<0.05; ***p*<0.01, ****p*<0.001).

**Fig. s3.** SEVI (50 μg/ml) was incubated with myricetin at various concentrations (50, 25, 12.5, 6.25, 3.13, 1.56, 0.78 and 0.39 μg/ml). The mixtures were washed one to five times with PBS buffer and centrifuged to remove soluble myricetin. The pellets were resuspended in the original volume of medium and mixed with CCR5-tropic HIV-1_SF162_ (**a**) or CXCR4-tropic HIV-1_NL4-3_ (**b**). The luciferase activities of the cultures were measured at 72 h post-infection. Average values (±SD) were calculated from triplicate measurements; the data shown here represent one representative trial of three independent experiments. One-way ANOVA with Dunnett’s post hoc multiple comparisons test was used to statistically analyze the differences between samples containing SEVI alone and samples containing SEVI and myricetin (**p<0.05; **p<0.01, ***p<0.001*).

**Fig. s4.** The total p24 antigens of the mixtures of myricetin at various concentrations and 100 ng/ml HIV-1 virions without SEVI fibrils were detected in parallel with virus pull-down assays under the exact same conditions. (**a**) HIV-1_SF162_ and (**b**) HIV-1_NL4-3_. Average values (±SD) were calculated from triplicate measurements, and the data represent one representative trial of three independent experiment.
